# Binding of the HSF-1 DNA-binding domain to multimeric *C. elegans* consensus HSEs is guided by cooperative interactions

**DOI:** 10.1038/s41598-022-12736-x

**Published:** 2022-05-28

**Authors:** Lukas Schmauder, Siyuan Sima, Amira Ben Hadj, Ricardo Cesar, Klaus Richter

**Affiliations:** 1 Center for Integrated Protein Research at the Department of Chemistry, Technische Universität München, Lichtenbergstr. 4, 85748 Garching, Germany; 2 Coriolis Pharma Research GmbH, Fraunhoferstraße 18b, 82152 Martinsried, Germany

**Keywords:** DNA, Transcription factors, Microarray analysis, Analytical biochemistry

## Abstract

The protein HSF-1 is the controlling transcription factor of the heat-shock response (HSR). Its binding to the heat-shock elements (HSEs) induces the strong upregulation of conserved heat-shock proteins, including Hsp70s, Hsp40s and small HSPs. Next to these commonly known HSPs, more than 4000 other HSEs are found in the promoter regions of *C. elegans* genes. In microarray experiments, few of the HSE-containing genes are specifically upregulated during the heat-shock response. Most of the 4000 HSE-containing genes instead are unaffected by elevated temperatures and coexpress with genes unrelated to the HSR. This is also the case for several genes related to the HSP chaperone system, like *dnj-12, dnj-13,* and *hsp-1*. Interestingly, several promoters of the dedicated HSR-genes, like *F44E5.4*p, *hsp-16.48*p or *hsp-16.2*p, contain extended HSEs in their promoter region, composed of four or five HSE-elements instead of the common trimeric HSEs. We here aim at understanding how HSF-1 interacts with the different promoter regions. To this end we purify the nematode HSF-1 DBD and investigate the interaction with DNA sequences containing these regions. EMSA assays suggest that the HSF-1 DBD interacts with most of these HSE-containing dsDNAs, but with different characteristics. We employ sedimentation analytical ultracentrifugation (SV-AUC) to determine stoichiometry, affinity, and cooperativity of HSF-1 DBD binding to these HSEs. Interestingly, most HSEs show cooperative binding of the HSF-1 DBD with up to five DBDs being bound. In most cases binding to the HSEs of inducible promoters is stronger, even though the consensus scores are not always higher. The observed high affinity of HSF-1 DBD to the non-inducible HSEs of *dnj-12*, suggests that constitutive expression may be supported from some promoter regions, a fact that is evident for this transcription factor, that is essential also under non-stress conditions.

## Introduction

The heat shock transcription factor (Hsf) is the essential transcriptional activator of the heat-shock response (HSR). It activates the genes of the classical HSR like Hsp70, small HSPs and Hsp40s^1–3^. Hsf proteins are further involved in many developmental processes, like embryonic placenta development^4^, female meiotic division^5^, and general transcription^6^. Hsf proteins are also reported as negative regulators of RNA polymerase II promoters and modulate protein homeostasis, cellular proliferation^7,8^ and the regulation of multicellular organism growth^9,10^. While these functions are governed by the binding of Hsf proteins to HSEs distributed throughout the genome, they also dependent on the chromatin accessibility^11^, as well as the general environment in which these Hsf proteins are activated^12,13^. In the mammalian genome several Hsf-like proteins are encoded that control individual sets of target genes. In nematodes, the expressed, full-length Hsf-like gene (HSF-1) is encoded, while another Hsf-homolog gene, termed *hsf-2*, is represented by the pseudogene Y53C10A.3. With HSF-1 likely being the sole expressed and functional homolog of the Hsf-proteins, the interaction between HSF-1 and the many detected HSEs can be investigated without the necessity to differentiate between several Hsf-proteins^14,15^.

Despite this simplicity, the regulation of the heat-shock response in nematodes is complex, being influenced by the age of the animal and active mostly in muscular and intestinal tissues^16^. Further, larvae up to the L2 stage show a reduced expression of the HSR in contrast to older larval forms. The aging adult then is characterized by lower inducibility of the HSR^17^. These differences imply a complex regulation of HSF-1 activity during aging, which is thought to ensure that the nematode’s reproductive phase is best protected from stressful events^18^. Beyond that, nematode HSF-1 is participating in the innate immune response by upregulating specific target genes and in aging, where HSF-1 cooperates with the transcription factor DAF-16^15,19–22^. The observation that several thousand of HSEs are present in the promoter regions throughout the nematode genome, even though the canonical HSR seems to be restricted to few genes^23,24^, is puzzling. Several of the canonical heat-shock proteins, like HSP-90 are not even heat-inducible in *C. elegans* and also the canonical Hsp40-like proteins are not upregulated strongly upon heat-shock^20,25^.

HSF-1 in nematodes is a protein of 671 amino acids. Like other Hsf proteins, HSF-1 consist of several conserved domains, including the N-terminal DNA-binding domain (DBD), an oligomerization domain and a carboxyl-terminal regulatory domain. Nematode HSF-1 further contains an 82-amino-acids extension of unknown function at its N-terminus. Under normal growth conditions Hsf proteins are monomeric and form cytosolic complexes with Hsp90 and Hsc70. This interaction prevents the trimerization and activation of Hsf proteins. Under heat-stress or other inducing conditions, Hsf proteins are released from the protecting chaperones and oligomerize. In most cases Hsf binds as a trimeric protein to the HSE-containing DNA sequences. The phosphorylation of Hsf proteins triggers the translocation to the nucleus and initiates the transcription^6,26–28^. Despite these regulatory events, the interaction of Hsf proteins with consensus dsDNA is observable also for the non-activated Hsf. In this respect, it is mostly unclear, how Hsf proteins distinguish the various HSE-containing target genes.

Here we focus on the DNA binding domain of HSF-1 from *C. elegans* and aim at resolving its interaction with differently regulated HSEs from the nematode genome. To this end we first define the HSE-containing genes that are strongly upregulated upon heat-shock. We then use HSE-containing dsDNA constructs from these HSE regions to investigate to what extent the interaction parameters of the HSF-1 DBD with dsDNA are influenced by HSEs of different sequences and structural organization.

## Material and methods

### Analysis of microarray data

Initially three microarray data sets investigating the heat-shocked (GSM62937, GSM62941, GSM62945) versus non-shocked condition (GSM62936, GSM62940, GSM62944), which can be obtained from the GEO microarray depository under the GSE2862 tag^31^, were used to identify genes with strong overexpression. Here, L2 stage *C. elegans* larvae were heat-shocked for 20 min at 33 °C, followed by a recovery at 20 °C for 40 min. As expected, and previously published^15^, the strongest upregulated genes were *hsp-16.1*, *hsp-16.48* and *F44E5.4* and their duplicated loci. Individual genes that show elevated expression under heat-shock conditions were determined. To obtain information on whether these genes commonly express together, genome-wide clique set analysis was performed as described before^29,30^. The heat-shock data sets were used together with the publicly available coexpression cliques. Altogether 307 cliques had been obtained before, with the largest clique containing 1200 genes and the smallest clique containing 6 genes and the publicly available information was used (www.richterlab.de/DataSets/ and https://github.com/klarichter/clusterEX_cliques_Celegans)^29,30^. We then used each of the three microarray replicates to assign their values to the genes in the coexpression cliques and analyzed those in respect to significant induction or repression as previously described for yeast and nematode expression studies^29,30^. As these heat-shock data and the clique set were both based on the GPL200 platform (Affymetrix C. elegans genome st-1.0), each Probe Set ID was represented by exactly one value in the described clique set. Analyses were performed for each replicate and average values for each clique were calculated to rank the cliques according to their average induction and p-values for induction significance as described^29,30^.

Given the complexity of the heat-shock response, we compared these data to other genome-wide expression data sets. As such a heat-shock time course defined by microarray data^32^ was investigated as well as heat-shock experiments based on RNA sequencing^15,17^ . The Subio64 software package 1.24.5853 (Subio Inc. Kagoshima, Japan) was used to derive annotated, normalized expression data from the publicly available SRA-files in cases where the annotated data were not available from GEO repository.

### HSE-detection in the promoter regions

HSE-detection in the promoter region 1000 bp upstream of the ATG was performed based on the PWM-models published for the human Hsf1’s DNA binding sequence^33^, which is represented by the following PWM pattern:

- A | 0.41 -3.20 1.13 1.05 -0.86 -0.55 -2.10 -1.61 -0.17 -1.28 0.58 -5.77 1.14 1.11 0.26
- C | -0.57 -5.19 -5.19 -3.58 0.84 0.73 0.02 0.34 1.14 0.06 -0.53 -5.19 -5.19 -5.19 -0.21
- G | 0.84 1.70 -3.11 -1.20 -0.18 0.37 -0.70 -4.50 -0.27 -2.63 0.58 1.71 -5.19 -1.83 0.23
- T | -5.77 -5.77 -5.77 -2.37 -0.08 -0.58 0.77 0.76 -2.40 0.79 -5.77 -5.77 -5.77 -5.77 -0.41

The 1000 bp promoter regions were obtained from Wormbase (www.wormbase.org)^34^ and searched with this HSF-1 consensus description. As recommended, a threshold level of 9 was used as lower limit for detection^35^. In several cases HSEs were detected in the same promoter and located only 5 bp from each other, which implies that the investigated HSE actually is a tetrameric HSE. If this pattern is observed a second time, a pentameric HSE-element was detected or in rare cases even larger arrays of HSEs were detected.

### HSF-1 fragmentation and purification

Fragmentation was performed based on hydropathy plots and expression tests, which indicated that fragments, which contained additional domains outside the DBD showed either very weak expression or insoluble expression and that full-length HSF-1 could also not be obtained in soluble amounts sufficient for biochemical analysis. Due to our plan to investigate the direct interaction with the DNA, we chose the isolated DBD as model protein for interaction analysis. Therefore, the N- and C-terminus of *C. elegans* HSF-1 were determined by comparing both hydropathy plots and sequence alignments of different Hsf proteins from diverse species. This yielded the fragment AA82-AA198 which was subcloned into the pGATE vector (HSF-1 DBD) and thereby fused with a GST-tag. A GST-trap column was used for purification and the GST-tag was cleaved off by TEV-protease before the HSF-1 DBD was further purified via ion exchange chromatography and size-exclusion chromatography (all columns from GE Healthcare, Chicago, USA). Purity was determined by SDS-PAGE and peptide fingerprinting using mass spectrometry on a Bruker ultra-flex III MALDI-TOF/TOF instrument (Bruker, Billerica, USA) was employed to confirm the identity of the protein.

### Circular dichroism spectroscopy

CD-spectroscopy on a Jasco J-715 was performed to obtain information on the structure and stability of the HSF-1 fragment. The folding state and the thermal stability of the expressed HSF-1 fragment was assessed at a concentration of 0.2 mg/mL in storage buffer (40 mM K_2_HPO_4_, 150 mM KCl). CD-spectra were recorded in the Far-UV region between 215 and 260 nm. To analyze the thermal stability of the fragment an unfolding transition was recorded at 220 nm in a temperature range between 25–95 °C.

### Thermal shift assays

The stability of the folded structure was analyzed with thermal shift assays in a Mx3005P qPCR cycler (Stratagene, La Jolla, USA). Thermal shift assays were performed at a protein concentration of 0.2 mg/mL after addition of SYPRO orange (Invitrogen, Waltham, USA) at a dilution of 1:1000. The total volume was adjusted to 20 µL with storage buffer. The emission of SYPRO orange is recorded by excitation at 470 nm at a wavelength of 570 nm to monitor the temperature induced transition in the temperature range of 25 to 95 °C.

### EMSA shift assays

ssDNA probes representing the promoter regions of *F44E5.5*, *hsp-16.2a*, *hsp16.2b*, *hsp-1*, *hsp-70*, *dnj-12 and dnj-13* were obtained from Eurofins MWG Biotech (Eurofins MWG Biotech, Ebersberg, Germany). An equal amount of forward and reverse complementary strand was incubated at 95 °C and aligned at room temperature. The interaction between these dsDNAs and the HSF-1 DBD was then monitored by EMSA shift assays, by performing a native PAGE after DBD was added to the dsDNA. Gels were incubated in SYBR green for DNA detection and analyzed in a Typhoon Fluorescence Scanner (GE Healthcare, Chicago, USA) using the Alexa Fluor filter at 532 nm and stained with Coomassie for visualization of the protein complex.

### Analytical ultracentrifugation and determination of species distributions

Analytical ultracentrifugation was performed in a ProteomeLab XL-A analytical ultracentrifuge (Beckman-Coutler, Brea, USA) to determine the binding of HSF-1 DBD to dsDNA sequences. To this end single strand DNA sequences from different promoter regions of the same length were mixed with equal amounts of their complement strand in storage buffer, heated up to 95 °C and then cooled to RT to generate the double stranded DNA product that represents the promoter region. HSF-1 DBD was added to 1.5 µM dsDNA at different concentrations (0 µM, 2.25 µM, 4.5 µM, 7.5 µM, 10.5 µM, 15 µM and 22.5 µM) and the absorbance of these samples was detected in analytical ultracentrifugation sedimentation velocity experiments at 260 nm and 280 nm at 42,000 rpm.

Data analysis of individual samples was performed with UltraScan III Version 4.0 (https://ultrascan3.aucsolutions.com/)^36^. All experiments were analyzed with the 2DSA-IT model employing the same settings (s-value range from 0 to 10 and f/f0 range from 1 to 4). This way two species distributions were obtained for each experiment, one for the data at 280 nm and one for 260 nm. The complexity of these distributions did not allow a unanimous assignment of solutes to species, which suggests that for a unifying solution a further reduction in search space has to be enforced. A reduced model therefore contained only the most abundant species of the binding reaction (HSF-1 DBD, ssDNA, dsDNA, dsDNA + 1 HSF-1, dsDNA + 2 HSF-1, dsDNA + 3 HSF-1, dsDNA + 4 HSF-1 and dsDNA + 5 HSF-1) at defined s_20,w_ values. These values were known for HSF-1 DBD, dsDNA and ssDNA from control experiments, while the other species were estimated from a stepwise optimization of these values. Given that all DNA strands were of the same size, a unique value for the sedimentation coefficient (s_20,w_) of each assembly intermediate was assigned independent of the dsDNA used.

A custom grid model containing the species at the respective s_20,w_ values was developed in UltraScan III and used to fit all data sets again. RMSD values of the unconstrained fit and the custom grid constrained fit were compared to verify that the fit quality despite the constraints is acceptable and the species s_20,w_ values are sufficiently good estimates. To estimate the specific volume of each species and to confirm the MW of each obtained species the following equation was used:

$${\overline{\text{v}}}_{{\text{c}}} = \sum\limits_{{{\text{i}} = 1}}^{{\text{N}}} {{\text{f}}_{{\text{i}}} {\overline{\text{v}}}_{{\text{i}}} = {\text{f}}_{{\text{p}}} {\overline{\text{v}}}_{{\text{p}}} + \sum {{\text{f}}_{{{\text{np}}}} {\overline{\text{v}}}_{{{\text{np}}}} } } ,$$

Value pairs for D_20,w_ and s_20,w_ were estimated and the extinction coefficients, specific volumes and molecular weights were calculated for each species in the custom grid model.

### Estimation of interaction parameters for dsDNA-DBD interaction

Data analysis was finally performed using the species concentrations determined from UltraScan III in the first unconstrained 2DSA-IT analysis and data fitting was based on previously developed models. The fitting function was modified from an Origin DLL-file developed originally for the interaction of two proteins (PPH-5 and HSP-90)^37^ to now describe the five-step binding process. Fitting was performed in analogy to the Nelder-Mead implementation for C# accessible at https://docs.microsoft.com/de-de/archive/msdn-magazine/2013/june/test-run-amoeba-method-optimization-using-csharp. Employing this function, K_D_-values for each step could be estimated. To this end, detected species absorbance was converted to species concentrations by employing the estimated extinction coefficients and fitting was performed globally for all species at both wavelengths. In few cases, especially where binding was very weak, RMSD values were almost exclusively influenced by the free ligand concentration. Under these conditions a weighing factor of 0.3 or 0.1 was applied to the free HSF-1 concentration to give more relevance to the other species M, ML1, ML2, ML3, ML4 and ML5. Cooperativity was observed, if K_D_-values for later assembly steps showed higher affinity than K_D_-values for early binding steps. Despite the constrained model the obtained K_D_-values contain large error intervals and are therefore considered as estimations due to the complexity of the binding events and the differences within the individual binding sites on the DNA.

### ChIPseq-data analysis

ChIPseq data as available from the GEO repository were obtained as bedgraph-files^17^. Bedgraph files were searched to retrieve the values for specified regions and the reads identified in HSF-1 IP under various conditions were summarized in Excel to display the regions relevant for the genes of interest.

## Results

### The heat-shock response is represented by a small set of genes in *C. elegans*

We initially aimed at identifying those HSE-containing promoters that are most strongly upregulated under heat-stress conditions. Given that the HSR is complex in nematodes we used data from several heat-shock studies based on microarray and RNAseq analysis. Besides defining the individual genes, which are induced upon heat-shock in the different experiments, we also tested, whether the differently regulated genes are enriched in one or more of the 307 *C. elegans* coexpression cliques. These groups of genes (or gene sets) were obtained based on coexpression analysis of more than 2000 microarray experiments recently^30^ and found to contain many coexpressing tissue specific, phenotype specific and GO-term specific gene sets.

**Figure 1:**
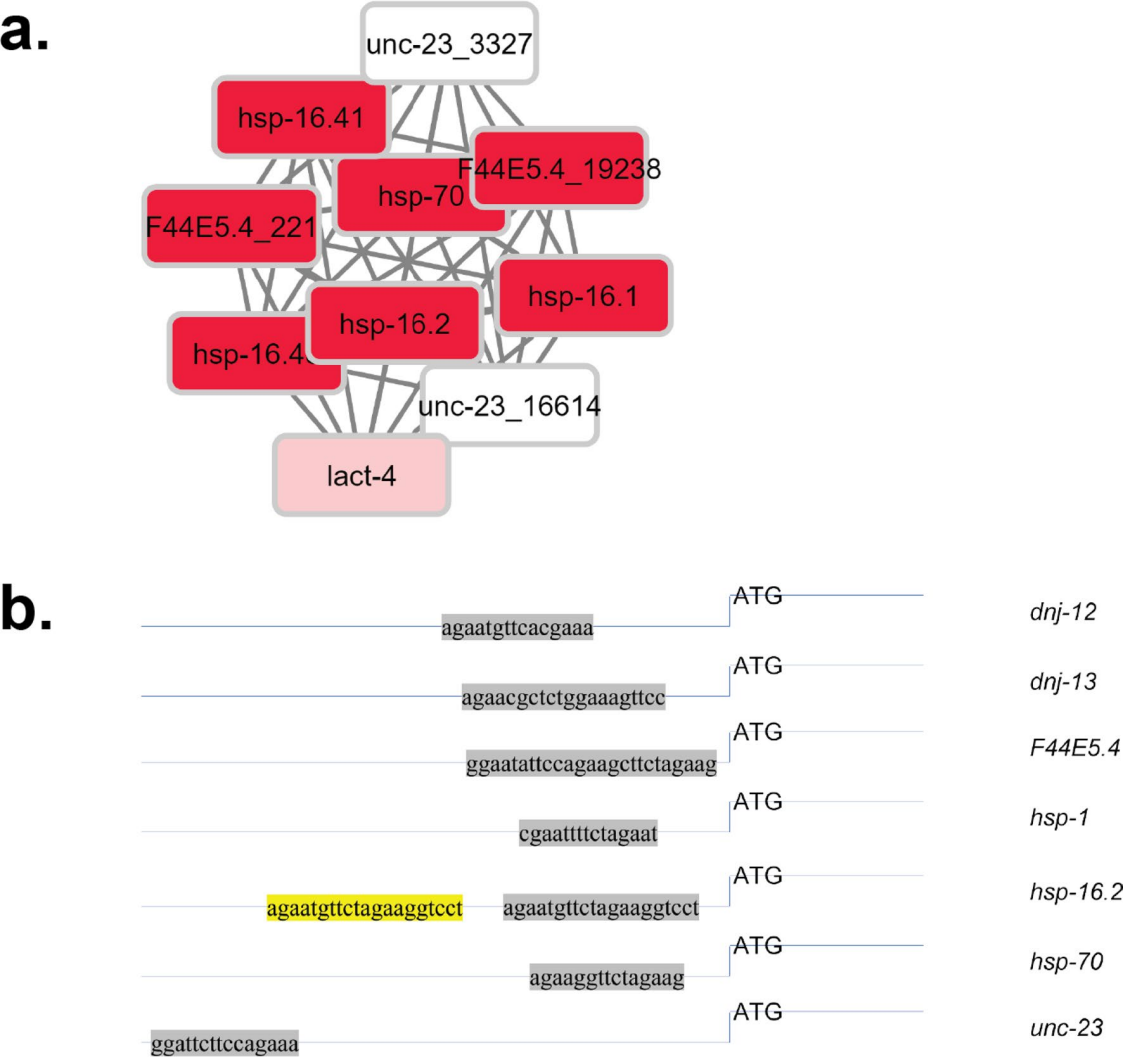
Determined highly induced coexpression cluster and utilized promoter regions. (**a**) The highly induced clique of the genome clustered in 307 expression groups derived from public expression experiments. The color code reflects the heat-shock response as determined by Wang et al. (GSE2862)^31^. The highly induced cluster is enlarged for better visualization, while the whole clique map of the genome can be found in Supplemental Fig. 1. (**b**) Promoter region of inducible and non-inducible chaperones, relative to the ATG start codon.

To initially define heat shock inducible and non-inducible HSE-sites in the promoter regions from the experiment series GSE2862^31^, we defined the gene sets (or cliques) that represent the heat-shock response under these conditions (L2 larvae, 20 min 33 °C followed by recovery period of 40 min). To this end, we used the method previously described^30^ to determine significant coregulation units responding to heat shock. The procedure searches the 307 predefined coexpression cliques and identifies those with significant expression changes. In all three replicates of GSE2862^31^ in particular one gene set out of the 307 cliques was highly induced (log_2_ > 2), the clique termed hsp-16.2-F44E5.4_19238, which contains the well described heat-shock genes *hsp-16.1*, *hsp-16.2*, *hsp-16.48*, *hsp-16.41*, *hsp-70*, *F44E5.4* and *F44E5.5* and in addition *unc-23* and *lact-4* (replicate 1 in Fig. 1a, Summary of the three replicates in Table 1, whole genome clustered in Supplemental Fig. 1). While *unc-23* and *lact-4* were not significantly upregulated in the three microarray experiments, the other genes of this coregulation clique are highly induced so that the hsp-16.2-F44E5.4_19238 gene set stands out with a 4.2-fold average induction (Table 2). Several canonical chaperones, like *dnj-12* (two probes in cliques cdc-42_17192-rab-5_18073 and srj-42-srw-113), *dnj-13* (two probes in cliques unc-116_2109-zfp-1_3976 and tars-1-AFFX-r2-3026-5_at) and the constitutively expressed Hsc70-homolog *hsp-1* (clique dld-1-skn-1_16701) are not part of the HSR-clique hsp-16.2-F44E5.4_19238 and we individually tested their induction to confirm that they are indeed not coregulated with the induced heat-shock proteins (Table 2). As we find them them not upregulated in either of the replicates, the assignment to other coexpression cliques seems justified..

**Table 1:** Cliques identified as significantly up- or downregulated in the heat-shock experiments of Wang et al. (GSE2862)^31^.

Clique number	Clique name	Clique size	Genes with signal in array	Average log_2_	STD log2
268	hsp-16.2-F44E5.4_19238	10	10	2.06	0.37
5	Y73B3A.2_1097-Y73B3A.2_1130	17	2	0.33	0.71
104	C10A4.6-C01A2.6	16	16	0.27	0.14
291	fbxc-6-F09C6.1	8	8	0.24	0.36
163	R05H5.4-F22D6.9_20949	5	5	0.22	0.27
235	C10C5.3-C10C5.5	6	6	0.22	0.20
241	fbxa-154_11499-Y44A6D.1	74	67	0.22	0.12
250	K12H6.6_1575-C23H4.6	6	6	0.19	0.33
80	C17H1.6-C17H1.13	76	75	0.19	0.13
169	C33H5.1-F49C12.2	11	11	0.18	0.36

The average values for the three replicate experiments are shown together with the standard deviation and the clique size.

**Table 2:** Significantly enriched genes and their clique assignment, or number of HSEs in the promoter region of the included genes.

Gene name	Average expression change log_2_	STD	Member of clique	Controlled by HSEs [number]
*hsp-16.41*	4.33964945	0.47257324	hsp-16.2-F44E5.4_19238	7
*hsp-16.1*	3.37025306	0.36880836	hsp-16.2-F44E5.4_19238	7
*hsp-16.2*	3.24598022	0.57114605	hsp-16.2-F44E5.4_19238	7
*F44E5.4_221*	2.43535939	0.6531605	hsp-16.2-F44E5.4_19238	10
*hsp-16.48*	2.43449799	0.26151971	hsp-16.2-F44E5.4_19238	7
*F44E5.4_19238*	2.42823859	0.70781857	hsp-16.2-F44E5.4_19238	10
*C27D6.1*	2.17492883	0.66776873	176662_at-Y53F4B.16	–
*fbxb-22*	2.1705405	1.27504799	sdz-10-fbxb-62	–
*math-6*	2.12527449	0.85966164	srj-21-srh-32	–
*F20B6.7*	1.95293402	0.75883293	tub-2_4713-daf-2_5288	–
*C15B12.3*	1.87784369	0.59022648	172183_at-176110_at	–
*Y6B3B.2*	1.79628476	1.35642504	183712_at-srbc-74_8864	–
*F28A10.9*	1.7697348	0.4485698	srj-42-srw-113	–
*C01B4.2*	1.74098252	0.42244849	srj-21-srh-32	–
*str-243*	1.72570157	0.42783197	sre-33-ZK1025.1_8337	–
*hsp-70*	1.69538497	0.43818876	hsp-16.2-F44E5.4_19238	3
*C07A9.5_18941*	1.68623723	1.47072224	F28H1.5-T05D4.5	–
*Y16E11A.2*	1.67500495	0.73573836	fbxa-154_11499-Y44A6D.1	12
*C31G12.3*	1.64731562	1.2334763	srj-42-srw-113	–
*F07B7.8_11449*	1.62495315	1.43400867	183712_at-srbc-74_8864	–
*clec-142*	1.61733446	1.76090815	F28H1.5-T05D4.5	–
*srh-258*	1.59055877	0.53259038	srj-42-srw-113	–
*scl-19*	1.59004684	1.03454503	srj-42-srw-113	3
*Y68A4B.3*	1.58552192	1.18455696	C33H5.1-F49C12.2	–
*clec-20*	1.58507895	0.97920764	sre-33-ZK1025.1_8337	–
*wrt-7*	1.58082879	0.50613839	grl-19-C13A2.4	–
*C04H5.7*	1.56608678	0.85708337	C46C2.5_15926-W03F11.1	–
*fbxb-118*	1.56221989	0.81840697	sdz-10-fbxb-62	–
*pqn-16*	1.5514195	1.07311037	srj-42-srw-113	–
*srh-154_13942*	1.53974391	1.00629988	srj-21-srh-32	2
…				
*lact-4*	0.48976226	0.47729707	hsp-16.2-F44E5.4_19238	–
*dnj-12_21104*	0.2791495	0.14801702	cdc-42_17192-rab-5_18073	3
*hsp-1*	0.23010296	0.59319525	dld-1-skn-1_16701	6
*unc-23_16614*	0.0047227	0.216626	hsp-16.2-F44E5.4_19238	6
*unc-23_3327*	− 0.07377314	0.30315485	hsp-16.2-F44E5.4_19238	6
*dnj-13_17082*	− 0.41124351	0.31596264	tars-1-AFFX-r2-3026-5_at	7
*dnj-12_3674*	− 0.49049134	0.41234554	srj-42-srw-113	3
*dnj-13_3319*	− 0.51080312	0.14089521	unc-116_2109-zfp-1_3976	7

Individual induced genes are shown and their association to cliques. Further the number of HSEs modules that can be found within 1000 bp of their upstream promoter sequence.

### Nematode HSEs vary widely in size and co-expression clique affiliation

We aimed at understanding, whether different affinities of the heat-shock transcription factor HSF-1 for the promoter sequences can be observed. Previous reports had highlighted that large number of HSEs can be found in the nematode genome^24,38^. Most of these genes are not induced in the heat-shock experiment investigated here. To obtain the HSEs of the genes of interest we searched the 1000 bp promoter regions of all genes of *C. elegans*. We identified 4120 HSE in genes, which contain a consensus sequence for HSF-1 in their promoter region. Despite not being induced upon heat-shock, several genes related to the chaperone system were found to contain HSE-like sequences in their promoter region, like *dnj-12*, *dnj-13,* and *hsp-1*. We then compared the sequence and structure of the HSEs in the promoter region of the chaperone proteins. Here, several promoters in the HSR-cluster contain more HSEs than the usually expected trimeric DNA-binding sequence, like *hsp-16.2a* and *F44E5.4*, which contain four or five HSF-1 binding sites in close vicinity (Fig. 1b).

### Heat-shock inducibility varies with the employed stress conditions

We used data from other heat-shock experiments—performed with RNAseq—to see, whether these chaperones and heat-shock proteins are induced with the same pattern. In these RNAseq experiments analysis had been performed in young adults and L2 larvae with and without a heat-shock exposure. In the experiment performed by Brunquell et al.^15^, a very similar set of genes was induced upon heat-shock and likewise only one coexpression clique out of the 307 was found to be significantly upregulated, the clique hsp-16.2-F44E5.4_19238. Concomitantly the chaperone genes also represent the strongest upregulated genes on the single-gene basis (Table 3, Supplemental Fig. 2a). This also was observed in the second RNAseq experiment performed by Li et al.^17^ in L2 and young adult larvae (Table 4, Supplemental Fig. 2b).

**Table 3:** Significantly enriched genes in the heat-shock experiments of Brunquell et al*.*^15^.

GeneName	Mean_noHS	Mean_HS	LOG_Ratio
F44E5.5	9.747	1706.353	7.452
F44E5.4	9.781	1703.300	7.444
R11A5.3	0.478	63.026	7.044
hsp-16.2	5.804	754.015	7.021
hsp-16.41	8.159	944.460	6.855
hsp-70	1.577	154.207	6.612
nspe-2	0.459	42.666	6.539
hsp-16.11	24.686	2280.832	6.530
hsp-16.1	27.345	2520.684	6.526
nspe-1	1.575	125.482	6.316
MTCE.8	0.754	53.406	6.147
hsp-16.49	29.527	1910.189	6.016
hsp-16.48	28.702	1855.322	6.014
K02F2.11	0.338	17.087	5.658
F26D10.24	0.330	12.838	5.282
MTCE.7	132.948	5111.687	5.265
ostf-4	0.303	6.745	4.477
21ur-14804	3.447	65.291	4.244
21ur-6528	3.807	67.278	4.143
col-84	0.346	4.882	3.819
Y17D7C.2	0.296	3.799	3.682
C13A2.12	0.341	4.320	3.663
MTCE.17	0.383	4.713	3.622
21ur-14438	0.286	3.474	3.602
MTCE.27	0.291	3.410	3.549
rpr-1	0.000	17.693	Inf
21ur-8810	0.000	11.528	Inf
lfor-1	0.000	8.067	Inf
lfor-2	0.000	7.890	Inf
21ur-4739	0.000	10.792	Inf
T06E8.3	0.000	3.935	Inf
nspe-5	0.000	6.856	Inf
….			
unc-23	18.625	75.578	2.021
dnj-13	73.991	188.783	1.351
hsp-1	606.752	1026.459	0.758
dnj-12	106.633	84.553	-0.335

Individual induced genes are shown compared to their non-stressed state. To define, which genes are expressed, it is to be considered that the median count is 7.7 and 5.2 for the two conditions investigated.

**Table 4:** Significantly enriched genes in the heat-shock experiments of Li et al.^17^.

GeneName	Mean_noHS	Mean_HS	LOG_Ratio
hsp-16.41	1.139	7114.820	12.609
hsp-16.2	1.414	7361.540	12.346
hsp-70	0.188	558.244	11.539
R11A5.3	0.810	1052.930	10.344
ZC21.10	1.134	378.419	8.382
R107.5	30.439	3524.790	6.855
C25F9.2	0.068	5.761	6.411
dct-8	0.483	32.069	6.054
nurf-1	63.938	4236.550	6.050
fbxb-72	0.122	7.221	5.886
Y71G12B.18	0.153	8.825	5.851
scp-1	15.798	863.127	5.772
F53A9.2	1.313	64.584	5.620
tts-1	9.980	446.779	5.484
Y43F8B.2	63.126	2546.830	5.334
dpy-17	2.135	78.616	5.202
Y38H6C.8	1.134	33.264	4.875
col-106	0.049	1.414	4.852
bath-34	0.441	12.651	4.843
mtl-1	1.301	35.785	4.782
nspc-12	0.568	15.630	4.782
R05D7.2	0.049	1.308	4.741
D2045.5	0.045	1.123	4.646
ugt-14	0.275	6.485	4.561
C30E1.9	139.027	3245.950	4.545
C01G12.14	0	74.4564	Inf
C09B8.12	0	14.777	Inf
C11G10.11	0	16.9277	Inf
C15H7.8	0	14.8235	Inf
C16A3.15	0	19.6119	Inf
C17H12.34	0	16.3819	Inf
C25E10.14	0	1.71674	Inf
….			
unc-23	52.588	326.964	2.636
dnj-13	161.747	792.283	2.292
hsp-1	1050.010	2194.130	1.063
dnj-12	217.845*	232.39*	0.093

Individual induced genes are shown compared to their non-stressed state. *The gene *dnj-12* was not included in the available final tables and therefore was calculated based on the raw data. The raw data values were divided by 40 to achieve a similar level of normalization as for the other genes. Raw data mean prior to normalization for all genes is 80 versus 83, thus substantially lower compared to the values of 8713.66 and 9295.77 found for *dnj-12* prior to normalization.

We inspected one other experiment^32^, which had determined a time course of the heat-shock response, to investigate whether further genes get differentially expressed after prolonged incubation at the heat-shock temperature. At the shortest incubation time, hsp-16.2-F44E5.4_19238 was the dominant differentially expressed gene set and the chaperones in this clique were the genes with the strongest expression changes (Table 5, Supplemental Fig. 2c, Clique set in Supplemental Fig. 3a). This changes with longer exposure times and after 720 min of heat-shock several cliques are differentially expressed representing gene groups from very different processes and tissue specific expression (Table 5, Supplemental Fig. 2d, Clique set in Supplemental Fig. 3b). The cliques identified differ in their kinetics to heat-stress, in that most are not substantially affected at the shortest heat-exposure (30 min), but get affected starting from 60 min incubation time (Supplemental Fig. 4). Of the genes expressed under the harshest conditions, only few contain HSEs in their promoter region and even under those conditions *dnj-12*, *dnj-13* and *hsp-1* are only weakly changing their expression levels, while the heat-shock genes grouped in hsp-16.2-F44E5.4_19238 are highly elevated at all time points. Therefore, we consider these HSE-regulated genes to be “heat-inducible” while *dnj-12*, *dnj-13* and *hsp-1* represent genes that change their expression more weakly under heat-shock, despite HSE-sequences in the promoter region. *unc-23*, despite having been assigned to the HSR coexpression clique hsp-16.2-F44E5.4_19238 by the global coregulation analysis, also is upregulated weaker compared to the small heat-shock proteins and the Hsp70s.

**Table 5:** Significantly enriched genes in the heat-shock experiments of Jovic et al.^32^.

Gene name	0 min level	30 min-0 min	60 min-0 min	120 min-0 min	180 min-0 min	240 min-0 min	360 min-0 min	480 min-0 min	720 min-0 min
hsp-16.41	9.36	15.27	15.45	15.12	16.79	17.21	16.50	16.20	16.11
hsp-16.2	8.74	14.25	14.48	14.43	16.22	16.84	15.60	15.39	15.15
F44E5.5	11.51	16.72	16.80	16.42	17.52	18.09	17.20	16.65	16.09
T27E4.8	10.31	15.48	15.85	15.43	16.51	17.23	16.50	15.95	15.79
T27E4.3	9.31	14.32	14.13	14.15	15.50	16.20	15.42	15.34	15.17
hsp-16.41	8.71	13.15	13.59	13.48	15.26	15.79	14.43	14.68	14.71
hsp-70	10.35	13.92	15.69	14.98	16.67	16.95	16.33	15.88	15.34
F44E5.5	10.54	13.77	14.37	14.35	15.57	16.03	15.62	15.72	15.52
skr-15	6.19	9.15	6.65	6.91	6.08	6.63	6.24	6.37	6.59
C18A3.5	7.57	10.41	8.24	8.68	8.39	8.81	8.60	8.90	8.95
T10D4.2	6.67	9.51	6.61	6.97	6.23	6.95	6.47	6.78	6.93
M117.5	6.34	8.58	6.42	6.81	6.44	6.71	6.23	6.55	6.58
nurf-1	12.27	14.36	16.72	15.82	17.33	17.13	16.90	16.70	15.92
ZK616.3	9.33	11.33	9.21	9.77	9.64	9.82	9.34	9.58	9.80
BJ140948	6.60	8.57	10.30	10.43	11.89	12.23	11.90	11.70	11.73
nurf-1	11.41	13.32	16.12	14.92	16.35	16.56	16.32	16.17	15.24
C08G5.7	6.00	7.83	6.14	6.30	5.85	6.22	5.82	6.08	5.89
F19B2.5	11.62	13.42	14.92	14.34	15.32	15.48	14.65	14.68	14.53
C25F9.6	7.81	9.54	10.97	10.42	11.38	11.59	11.10	11.52	10.97
aip-1	10.29	12.01	13.83	13.13	14.19	14.23	13.48	13.23	12.92
F59C12.4	11.51	13.22	13.43	13.69	15.84	15.26	15.69	15.99	15.87
F59C12.4	11.84	13.52	13.89	14.04	16.10	15.62	16.03	16.37	16.14
Y94H6A.10	11.96	13.63	14.01	14.20	15.21	15.11	14.86	15.03	14.94
F59C12.4	11.99	13.61	14.07	14.20	16.19	15.75	16.17	16.61	16.37
daf-21	11.88	13.46	14.18	14.07	14.54	14.81	15.13	14.96	14.93
…									
R11A5.3	9.21	9.96	14.32	13.43	15.87	15.47	15.68	14.96	14.62
dnj-13	10.58	12.02	12.77	12.15	12.43	12.56	11.63	11.35	11.39
unc-23	9.66	11.09	11.99	11.42	12.19	12.32	11.48	11.70	11.51
dnj-12	7.43	8.60	8.44	8.49	8.83	9.04	9.01	9.36	9.31
hsp-1	15.13	15.71	16.56	15.87	15.70	16.03	15.60	15.30	15.16

Individual induced genes are shown for 30 min, and 720 min of heat-shock compared to their non-stressed state.

### The isolated DBD of HSF-1 shows affinity to the* F44E5.4* inducible promoter

**Figure 2:**
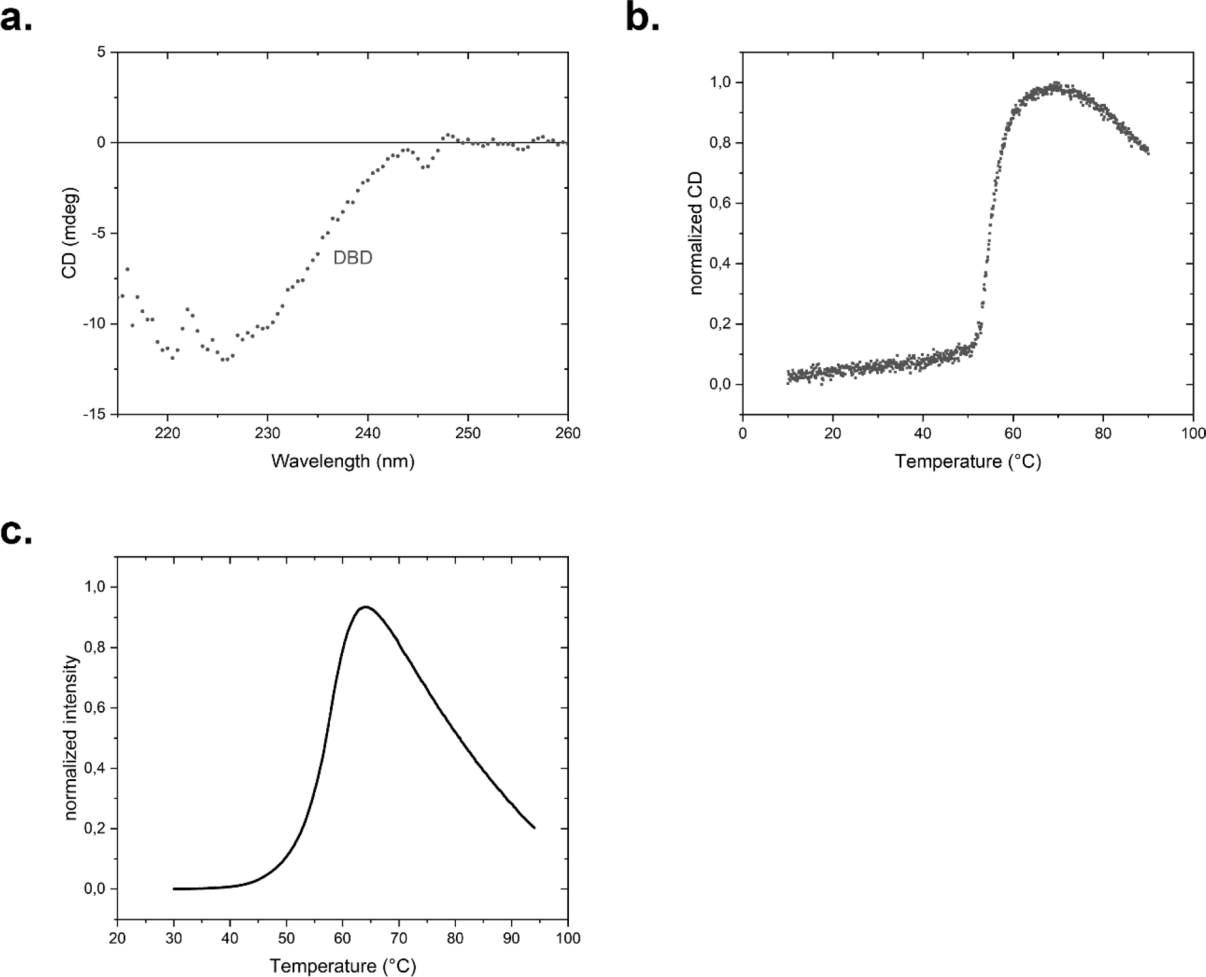
Structure and stability of HSF-1 DBD fragment 82–198. (**a**) CD-spectroscopy, (**b**) unfolding, determined by a thermal transition with CD-spectroscopy and (**c**) unfolding, determined by a thermal shift assay.

To test, to what extent binding differences correlate with expression differences and structural differences of the HSE we set out to determine in vitro, how the interaction of HSF-1 DBD is at these differently structured HSEs. To this end the isolated DNA binding domain of nematode HSF-1 was purified, containing the DBD and omitting the nematode-specific sequences at the N-terminus and the further regulatory domains at the C-terminus. The structure of the purified DNA-binding domain was investigated by far-UV CD-spectroscopy. The spectra revealed a mostly α-helical structure (Fig. 2a). To confirm the stability of the domain, we performed a thermal transition in the Far-UV CD-range and obtained a temperature midpoint of the unfolding transition of 55 °C (Fig. 2b). We also performed a stability investigation employing the TSA assay, where no obvious differences were observed regarding the melting point (Fig. 2c). Thus, all spectroscopic methods imply that the isolated DNA-binding domain of *C. elegans* HSF-1 is a stable and structured protein.

dsDNA probes were then generated by us from the heat-shock responsive cluster, in order to gain a better insight into the differential expression form the chaperone-gene derived HSEs. *F44E5.4* features a high consensus score pentameric site, both *hsp-70* and *unc-23* consist of only one trimeric site, while *hsp-16.2* has a high consensus score tetrameric site plus an additional trimeric site. Probes of equal length were also made for *hsp-1, dnj12* (trimeric HSE-site) and *dnj-13* (tetrameric site) representing the non-induced heat-shock related proteins. Since both sequence and position in the promoter region of the following genes are identical the probe for *F44E5.4* also represents *F44E5.5*, while *hsp-16.2* represents *hsp-16.11*, *hsp-16.41*, *hsp-16.48*. The sequences of the probes were obtained from the respective promoter regions. Here only HSEs were considered that locate within 1000 bps upstream of the starting point of transcription (Table 6). *F44E5.4*p contains more HSEs in its sequence than synthesized in this study (comparison of the promoter regions), but here likewise the probes with the highest consensus score were synthesized.

**Table 6:** HSE-containing probes designed from the promoter sequences of chaperone genes and used in the binding studies.

	Fwd	Rev	Trimeric consensus scores	Orien-tation	Start position relative to ATG	Promoter location			ORF location, orientation
DNJ-12	aaaagtgtcgagaatgttcacgaaaaatcgttaga	tctaacgatttttcgtgaacattctcgacactttt	9.66	Fwd	− 221	chrI, 14,756,377–14,757,376	14,756,377	14,757,376	chrI, 14,756,842–14,758,772
DNJ-13	agtaaatagaacgctctggaaagttccgcactctt	aagagtgcggaactttccagagcgttctatttact	13.01,	Fwd,	− 205	chrII, 11,544,000–11,547,000			chrII, 11,545,609–11,547,273
			9.1	Rev					+
F44E5.4 (also *F44E5.5*)	gcagtggaatattccagaagcttctagaagaagtt	aacttcttctagaagcttctggaatattccactgc	11.59,	Fwd, Rev, Fwd	− 124	chrII, 11,757,500–11,760,300			11,757,016–11,761,764
			12.43,						+ ,–
			13.07						
HSP-1	tgacgaaattcgaattttctagaatcccgccacgc	gcgtggcgggattctagaaaattcgaatttcgtca	10.24	Fwd	− 150	chrIV, 17,277,000–17,281,000			chrIV, 17,278,910–17,281,289
Hsp-16.2a	gccttacagaatgttctagaaggtcctagatgcat	atgcatctaggaccttctagaacattctgtaaggc	9.41,	Rev,	− 117 (*hsp-16.49* and *hsp-16.41*)	chrV, 1,804,000–1,806,000			chrV, 1,804,268–1,804,799
			12.81	Fwd	− 253 (*hsp-16.11* and *hsp-16.2*)				–
Hsp-16.2b	acaagcagctcgaatgttctagaaaaaggtggaaa	tttccacctttttctagaacattcgagctgcttgt	11.86	Fwd	− 104	chrV, 1,804,000–1,806,000			chrV, 1,804,268–1,804,799
									–
Hsp-70	agtaaattgtagaaggttctagaagatgccagagg	cctctggcatcttctagaaccttctacaatttact	12.71	Fwd	− 112	chrI, 9,319,000–9,323,000			chrI, 9,320,350–9,322,501
									–
Unc-23	acggagcctcggattcttccagaaaattgagtctc	gagactcaattttctggaagaatccgaggctccgt	9.48	Fwd	^−^573	chrV, 8,935,000–8,939,000			chrV, 8,936,195–8,939,472
									+ , + , +

The designed probes originate from the 1000 bp promoter sequence and are positioned as indicated. The strand and anti-sense strand were synthesized and combined to give the promoter sequence able to bind HSEs.

### EMSA-assays imply differences between the chaperone-gene derived HSEs

**Figure 3:**
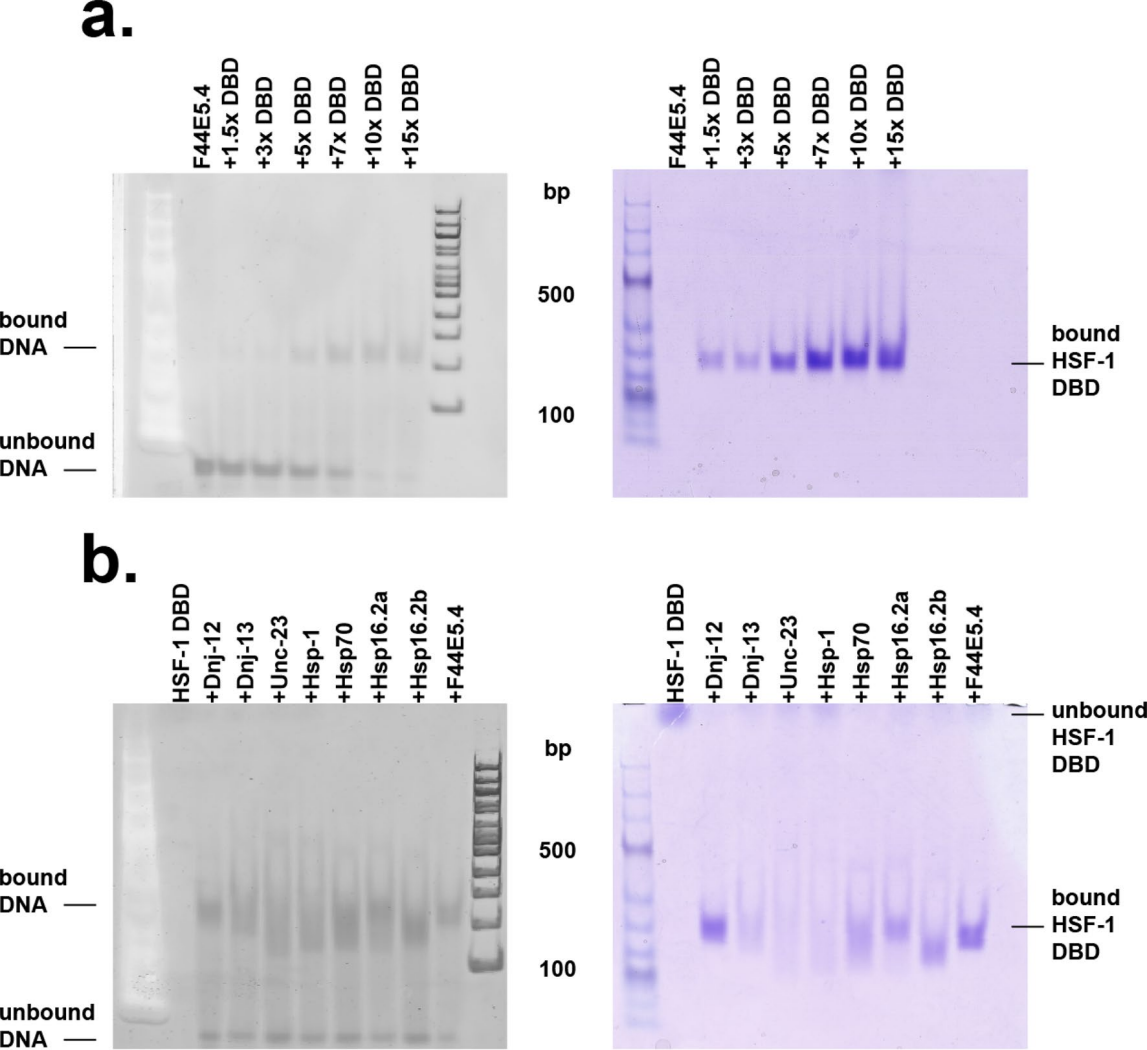
EMSA shift of the DNA-HSF-1 complex. The DNAs were stained with the DNA stain (left gel) whereas proteins were stained with Coomassie Blue (right gel). DNA-DBD binding occurs when both stained DNA and stained protein overlap, in comparison to each other. (**a**) Titration of HSF-1 DBD to the promotor F44E5.4, ranging from a 1,5–15-fold excess of HSF-1 DBD; (**b**) Comparison of selected DNA promotor sequences, each added to the HSF-1 DBD.

Electrophoretic-mobility shift assays (EMSA) were performed to test the interaction between purified HSF-1 DBD and dsDNAs (Fig. 3a). We set out to perform an initial binding analysis HSF-1 DBD to the promotor of *F44E5.4*, which also contains the highest amount of HSEs compared to the promoters used in this study. To this end, we titrated the DBD of HSF-1 with concentrations ranging from 0–22.5 µM to 1.5 µM of promoter DNA, which represents a 15-fold molar excess at the highest concentration. Notably a saturated complex of protein and DNA was reached at a concentration of 10 µM HSF1-DBD, at which the complex bands could be observed on the Coomassie stained gel, while at the same time no further reduction in free DNA was visible. Following this initial analysis, we also tested the dsDNA probes of *hsp-70, hsp-1*, *hsp-16.2, unc-23, dnj-12* and *dnj-13* under the same conditions. 10 µM HSF-1 DBD was added to each probe to determine the formation of the respective protein-DNA complex (Fig. 3b), which showed depending on the probe used, a highly variable reduction in migration speed. While probes derived from the promoter of *dnj-13, unc-23* and *hsp-1* hardly showed any interaction with the DBD of HSF-1, *F44E5.4*, *hsp-70*, *hsp16.2* and *dnj-12* derived probes appeared to interact strongly, thereby forming intense bands with HSF-1 DBD, representing the dsDNA-protein complex. These results indicate that the HSF-1 DBD alone can interact with the different promoter-derived HSEs to a different extent.

### Analytical ultracentrifugation confirms the binding differences at the various HSE-sites

**Figure 4:**
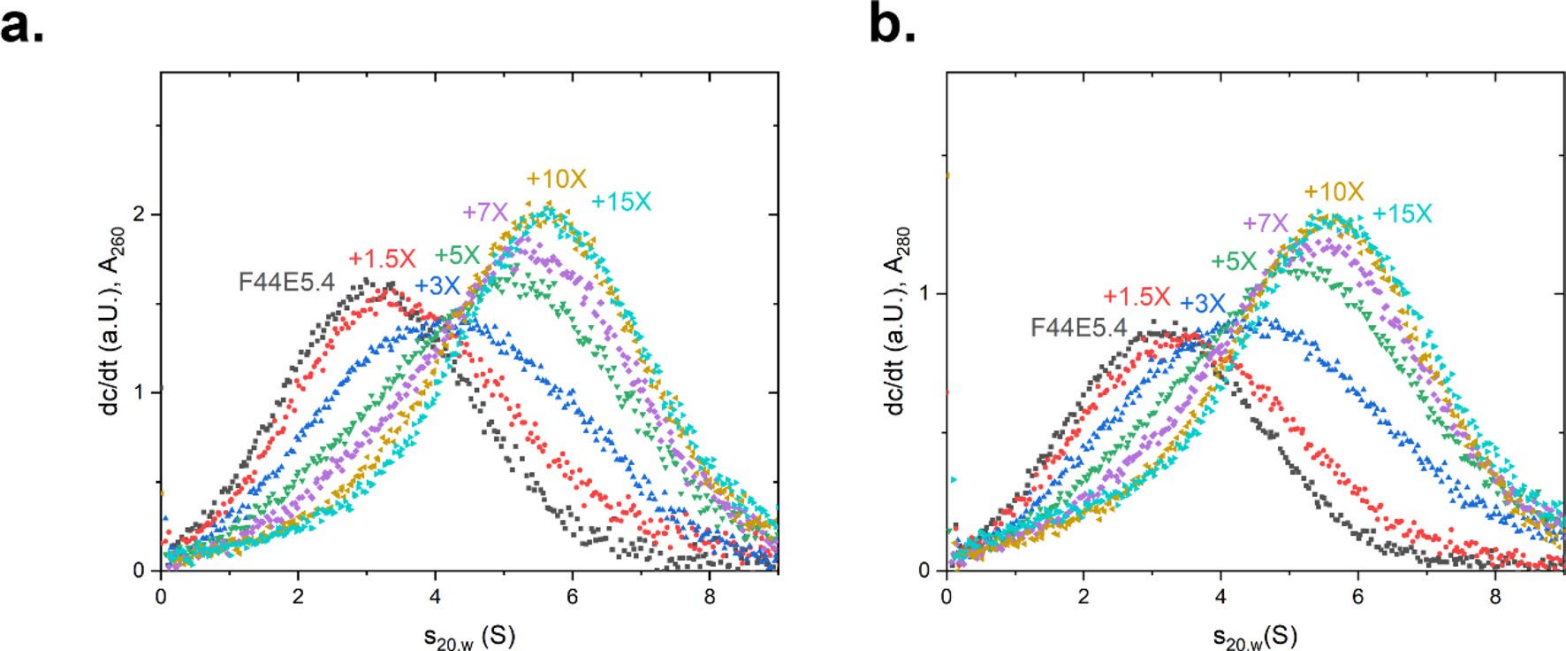
Analysis of interaction between promotor F44E5.4 and Hsf-1 DBD via titration in SV-AUC. HSF-1 DBD was titrated to the promoter F44E5.4 in concentrations ranging from a 0–15-fold excess. A shift to the right represents DNA-binding by the HSF-1 DBD. (**a**) dc/dt plot of the absorbance measured at 260 nm and for (**b**) at 280 nm at different concentrations of HSF-1 DBD, when added to 1.5 µM of HSE-containing DNA.

To unravel the interaction patterns, we performed SV-AUC under the condition employed for the gel-based assay. To this end, a titration with the DNA probe representing *F44E5.4*p was performed. Addition of HSF-1 DBD resulted in an increase in the sedimentation coefficient, indicating the binding of HSF-1 DBD to dsDNA (Fig. 4). In the titration, the progressive binding of HSF-1 DBD molecules increases the s_20,w_ of the main species and indicates further complex formation at higher protein:DNA ratios. The complex with *F44E5.4*p appears to reach a saturated level when a tenfold excess of HSF-1 DBD is added. At this point, the presence of remaining unbound HSF-1 DBD becomes visible, which is in agreement to the EMSA binding assay.

**Figure 5:**
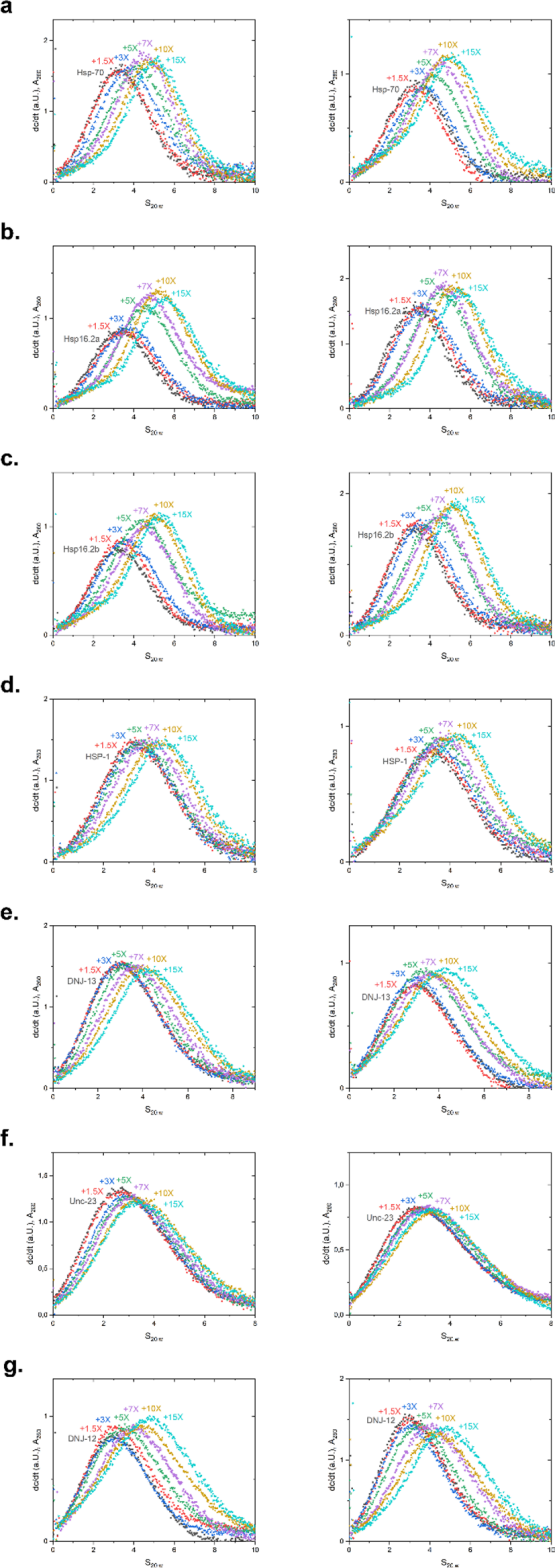
Analysis of interaction between selected promotors and Hsf-1 DBD via titration in SV-AUC. HSF-1 DBD was titrated to each promoter in concentrations ranging from a 0–15-fold excess. A shift to the right represents DNA-binding by the HSF-1 DBD. dc/dt plots of the absorbance measured at 260 nm (left panel) and at 280 nm (right panel). Respective promoters used: (**a**) HSP-70; (**b**) HSP16.2a; (**c**) HSP16.2 b; (**d**) HSP-1; (**e**) DNJ-13; (**f**) UNC-23; (**g**) DNJ-12.

Having investigated the promoter region with 5 potential binding sites, we tested, whether the promoter regions with less binding sites, show a similar response. Thus, the same approach was chosen for a DNA with only 3 binding sites derived from the promoter of *hsp-70*. Here the saturation point of the binding reaction was shifted to lower s_20,w_ values in both wavelength detection modes, suggesting that in this case less HSF-1 DBD molecules bind to the promoter (Fig. 5a). This behavior therefore appears to be a sequence-specific property. Further analog experiments were performed with all the other dsDNA strands and initially the highest s_20,w_ values were noted (Fig. 5b-g).

### SV-AUC fitting to defined species reveals potential differences in occupation of complex binding sites

**Figure 6:**
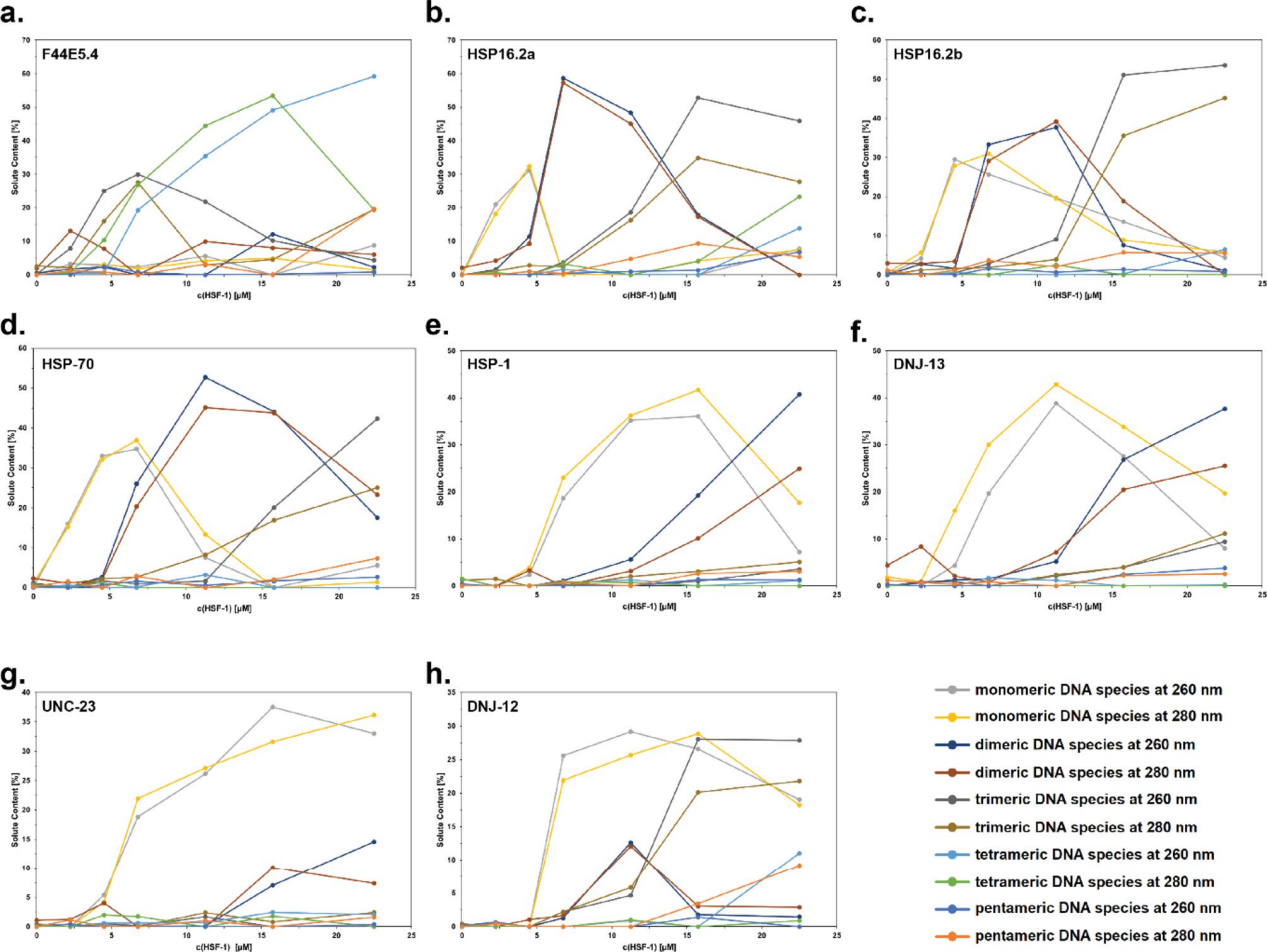
Partial concentration of each species in DNA-HSF-1 samples, derived via custom grid fitting. Selected promoter = (**a**) F44E5.4; (**b**) HSP16.2a; (**c**) HSP16.2b; (**d**) HSP-70; (**e**) HSP-1; (**f**) DNJ-13; (**g**) UNC-23; (**h**) DNJ-12.

The very weak interaction at several consistent—at least on a monomeric level—HSE sites, questions the independent interaction of monomeric units at these sites. UltraScan III was employed to analyze the data from these experiments and to obtain information on the binding equilibrium in solution. To this end we compared the general ability to fit the data with a very flexible model (2DSA-IT) and with a very constrained model, where a custom grid was designed containing one s_20,w_ value (Table 7) for each species to be considered (2DSA-CG-IT). This method reveals available free protein concentration dependent changes in complex species distributions and offers the opportunity to fit distributions of DNA/protein complex obtained directly from raw data to hypothetical species, thus, to obtain the concentration of each potential complex species and to describe the composition of the complex mixture in each sample. The comparison of RMSD values from the 2DSA-CG-IT fit of each complex species formed with different DNAs is shown in Fig. 6. It is very clear from these data that different assembly mechanisms are happening in different probes and different stoichiometries must be assumed. In the UltraScan III analysis, the higher order complexes are only populated when using larger HSEs and in all cases the buildup of the free HSF-1 DBD can be observed at the higher concentrations employed in each titration. Furthermore, almost no binding was observed for the constructs of *hsp-1, dnj-13,* and *unc-23.* (Fig. 6e, f and g).

**Table 7:** Parameters used for custom grid fitting approach.

	s_20,w_ (S)	E_260_ (M^−1^ cm^−1^)	E_280_ (M^−1^ cm^−1^)
HSF-1 DBD	1.75	8,388	13,980
dsDNA	3.15	563,198	304,431
ssDNA (residual from mixing)	1.75	355,400	192,108
dsDNA + 1 DBD	3.9	571,586	318,411
dsDNA + 2 DBD	4.65	579,974	332,391
dsDNA + 3 DBD	5.35	588,362	346,371
dsDNA + 4 DBD	5.95	596,750	360,351
dsDNA + 5 DBD	6.55	605,138	374,331

Generally applicable sedimentation coefficients were obtained for this model system, where HSEs are embedded into probes of identical size. Extinction coefficients are also shown. f/f0 was kept floating during the fit with the custom grid model, while the s_20,w_ was set to allow only the species mentioned in this table.

### Global fitting of stepwise binding models implies favorable cooperative action at second and third binding steps

We then set out to globally fit one titration to a predefined set of species, which is kept invariant throughout all the DNA probes analyzed. This is possible, as the dsDNA strands are of equal length and the binding sites are engineered to be in the middle of each dsDNA scaffold. Indeed, for each of the stronger binding species, the second binding step is exposing a lower dissociation constant compared to the first binding steps and similar relationships occur at the later binding steps at probes that harbor more than three binding sites. In fact, the four strongly interacting systems (*hsp-70*, *hsp-16.2a*, *hsp-16.2b* and *F44E5.4*) show a second binding step with submicromolar affinity, while the first binding step is weaker (Table 8). Thus, it is indeed to be expected that cooperative actions increase the binding affinity and interactions between the occupied binding sites modulate and potentially coordinate the binding of HSF-1 at these HSEs.

**Table 8:**
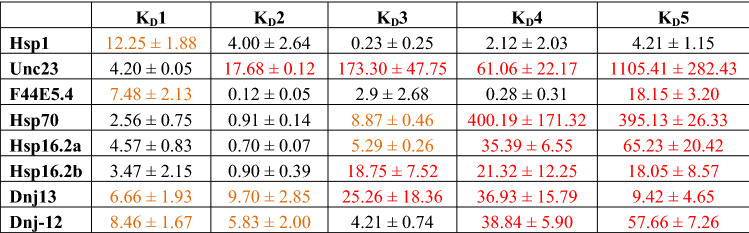
Calculated K_D_ values derived from SV-AUC fitting.

No color coding = cooperativity, orange color coding = weak cooperativity, red color coding = no cooperativity.

## Discussion

In the nematode genome there are 4120 HSEs, which contain HSF-1 binding consensus regions in the 500 bp upstream of their start codon. It is very surprising that despite these many HSF-1 regulated genes the canonical heat-shock response only represents a clique of 8 genes, 7 of which are regulated by HSF-1 binding promoter regions. Thus, the extent of regulation resulting from HSF-1’s actions is well beyond the induction of stress genes under stress conditions and reaches far into the normal growth cycle of the nematode under non-stressed conditions. The ability to resolve the clique membership based on coexpression analysis shows that also in larger organisms this approach may be successful and able to connect different cliques to different tissues and developmental states.

### Binding affinity, cooperativity and stoichiometry on complex promoter sequences

We here tested the binding of the HSF-1 DBD to some of the likely interacting promoter regions. From these studies we can find that the HSF-1 DBD alone can bind the HSE-regions originated from the genome with certain selectivity based on its affinity. Despite this, the affinities correlate to some extent with the calculated consensus score and with the inducibility of the respective gene. It is interesting to note, that despite the proposed trimeric binding mode, tetrameric and pentameric HSEs exist and that binding to those sites is driven by additional cooperativity. Among the probes we investigate in this study, the tetrameric and pentameric sites represent those, which are inducible upon heat-shock.

In general, the developed AUC assay to test the binding of several proteins to one DNA strand is very valuable in quantifying the binding events and may represent an opportunity to study the many interactions occurring on dsDNA with different binding sites for individual transcription factors. While the sedimentation coefficients for the custom grid are an assumption, they provide a rational to obtain stepwise binding information from the SV-AUC titration data. The absolute values of the obtained stepwise dissociation constants are to be used with care, but trends can be derived from these values with good confidence. The ability to resolve different intermediate assembly steps may be further increased by using direct interaction models for the fitting, but the stepwise procedure shown here already represents the chance to quantify these events. Nevertheless, the grouping of the genes into coexpression cliques, the identification of common transcription factors for these cliques and the analysis of binding events to the predicted transcription factor binding sites opens possibilities to gain further insight into the complex relationships leading to the spatio-temporal expression of genes during development and aging of *C. elegans*, or complex multi-step binding reactions in general.

### Correlation between binding and inducibility

Comparing the binding ability of HSF-1 to the promoter regions and the observed response to heat-stress may be far fetching, given that only the DBD of HSF-1 was studied and further regulation will surely come from the other regions of this complex protein. Nevertheless, for the strongest inducible genes, also the highest affinities are observed (*hsp-16.2*, *F44E4.5*, *hsp-70*), which are also in accordance with previous studies^15,17^.

**Figure 7:**
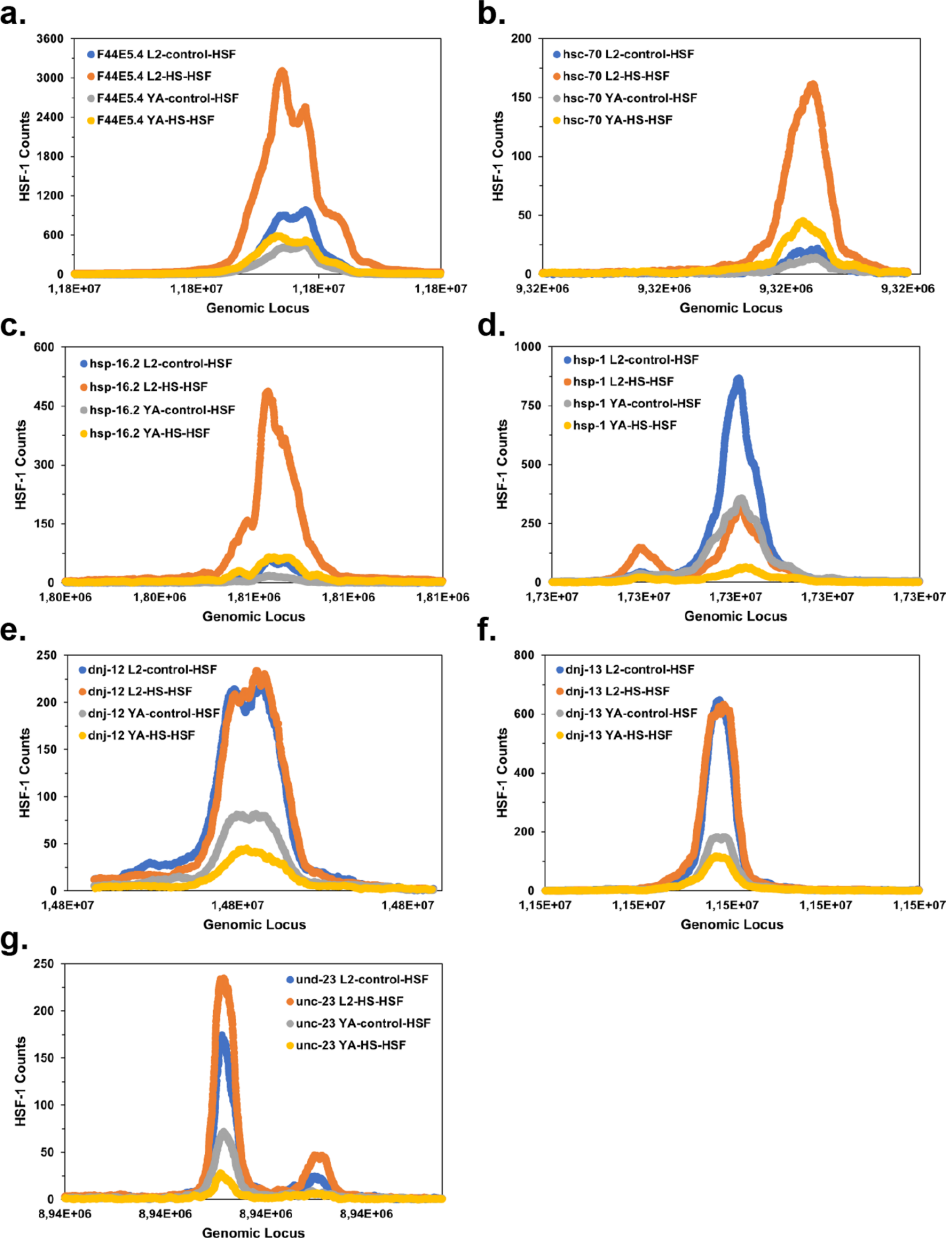
Promoter regions *of C. elegans* were investigated to identify the occupancy as determined by HSF-1 ChIPseq data of Li et al*.*^17^. Four experiments were compared based on the available data: Young adult with and without heat-shock and L2 larvae with and without heat-shock for all the promoter regions investigated here.

One exception among the probes studied here is *dnj-12*, which is only weakly inducible but well capable of binding to the HSF-1 DBD. Interestingly *dnj-12* is already at non-stressed conditions highly expressed, similarly to *hsp-1*. This can be derived from the relatively high number of RNAseq reads originating from these ORFs. Given the ubiquitous expression of this protein it might be envisioned that its binding to HSF-1 is constitutive, and the induced expression therefore is not increased upon heat-shock. Looking into publicly available ChIPseq data^17^ for the locations described here, some of these speculations can be tested. Indeed, for the genes coexpressed upon heat-shock, *hsp-16*, *F44E5.4* and *hsp-70* this can be confirmed (Fig. 7) and the inducibility from the promoters *F44E5.4/5*, *hsp-16.2* and *hsp-70* correlates well with increased occupancy of HSF-1 on the HSE-sites. Even for *unc-23* a slight increase in occupancy can be observed. This change at the promoter regions cannot be observed for the non-inducible probes. Here (*dnj-12*, *dnj-13* and *hsp-1*), HSF-1 sites are occupied in a similar or even reduced manner with and without heat-shock implying a constitutive expression and possible constitutive function of HSF-1 responsible for the high expression levels observed for these genes under stressed and non-stressed conditions. This logic may be relevant for several of the 4120 HSE-binding sites found in promoter regions. Despite the correlations observed, it is important to note, that the approach employed in this study solely considers the DBD of HSF-1 and that HSF-1 HSE binding in the cell is further regulated by other regulatory domains, oligomerization domains and posttranslational modifications, like phosphorylation^39,40^ and deacetylation^41^. Due to these limitations further studies with longer fragments or full-length protein will need to be performed to unravel the full relationship between promoter sequences and HSF-1 binding.

Therefore, the here applied approach shows the direct affinity of the unmodified DBD to the DNA, but will require adaptations, when used for the dsDNA binding analysis of full-length HSF-1 in the future.

## Supplementary Information

Supplementary Figures.

## Data Availability

All data are fully available without restriction.

## References

[1] Vihervaara A, Sistonen L (2014). HSF1 at a glance. J. Cell Sci..

[2] Skaggs HS (2007). HSF1-TPR interaction facilitates export of stress-induced HSP70 mRNA. J. Biol. Chem..

[3] Williams RS, Benjamin IJ (2000). Protective responses in the ischemic myocardium. J. Clin. Invest..

[4] Xiao X (1999). HSF1 is required for extra-embryonic development, postnatal growth and protection during inflammatory responses in mice. EMBO J..

[5] Kallio M (2002). Brain abnormalities, defective meiotic chromosome synapsis and female subfertility in HSF2 null mice. EMBO J..

[6] Kaitsuka T, Tomizawa K, Matsushita M (2011). Transformation of eEF1Bdelta into heat-shock response transcription factor by alternative splicing. EMBO Rep..

[7] Kubota H, Matsumoto S, Yokota S, Yanagi H, Yura T (1999). Transcriptional activation of mouse cytosolic chaperonin CCT subunit genes by heat shock factors HSF1 and HSF2. FEBS Lett..

[8] Cahill CM, Waterman WR, Xie Y, Auron PE, Calderwood SK (1996). Transcriptional repression of the prointerleukin 1beta gene by heat shock factor 1. J. Biol. Chem..

[9] Pirkkala L, Nykanen P, Sistonen L (2001). Roles of the heat shock transcription factors in regulation of the heat shock response and beyond. FASEB J..

[10] Gidalevitz, T., Prahlad, V., & Morimoto, R. I. The stress of protein misfolding: from single cells to multicellular organisms. *Cold Spring Harb Perspect Biol*10.1101/cshperspect.a009704 (2011). 10.1101/cshperspect.a009704 PMC3098679 21536706

[11] Guertin MJ, Lis JT (2010). Chromatin landscape dictates HSF binding to target DNA elements. PLoS Genet..

[12] Mendillo ML (2012). HSF1 drives a transcriptional program distinct from heat shock to support highly malignant human cancers. Cell.

[13] Li J, Labbadia J, Morimoto RI (2017). Rethinking HSF1 in stress, development, and organismal health. Trends Cell Biol..

[14] Joutsen, J. & Sistonen, L. Tailoring of proteostasis networks with heat shock factors. *Cold Spring Harb Perspect Biol* **11**, doi:10.1101/cshperspect.a034066 (2019). 10.1101/cshperspect.a034066 PMC6442201 30420555

[15] Brunquell J, Morris S, Lu Y, Cheng F, Westerheide SD (2016). The genome-wide role of HSF-1 in the regulation of gene expression in Caenorhabditis elegans. BMC Genomics.

[16] Guisbert E, Czyz DM, Richter K, McMullen PD, Morimoto RI (2013). Identification of a tissue-selective heat shock response regulatory network. PLoS Genet..

[17] Li J, Chauve L, Phelps G, Brielmann RM, Morimoto RI (2016). E2F coregulates an essential HSF developmental program that is distinct from the heat-shock response. Genes Dev..

[18] Labbadia J, Morimoto RI (2015). Repression of the heat shock response is a programmed event at the onset of reproduction. Mol. Cell.

[19] Hsu AL, Murphy CT, Kenyon C (2003). Regulation of aging and age-related disease by DAF-16 and heat-shock factor. Science.

[20] Morley JF, Morimoto RI (2004). Regulation of longevity in Caenorhabditis elegans by heat shock factor and molecular chaperones. Mol. Biol. Cell.

[21] Brunquell J, Snyder A, Cheng F, Westerheide SD (2017). HSF-1 is a regulator of miRNA expression in Caenorhabditis elegans. PLoS ONE.

[22] Ben-Zvi A, Miller EA, Morimoto RI (2009). Collapse of proteostasis represents an early molecular event in Caenorhabditis elegans aging. Proc. Natl. Acad. Sci. USA.

[23] Morimoto RI (2011). The heat shock response: systems biology of proteotoxic stress in aging and disease. Cold Spring Harb Symp. Quant. Biol..

[24] GuhaThakurta D (2002). Identification of a novel cis-regulatory element involved in the heat shock response in Caenorhabditis elegans using microarray gene expression and computational methods. Genome Res..

[25] Gaiser AM, Kaiser CJ, Haslbeck V, Richter K (2011). Downregulation of the Hsp90 system causes defects in muscle cells of Caenorhabditis elegans. PLoS ONE.

[26] Wang X (2006). Phosphorylation of HSF1 by MAPK-activated protein kinase 2 on serine 121, inhibits transcriptional activity and promotes HSP90 binding. J. Biol. Chem..

[27] Xing H, Mayhew CN, Cullen KE, Park-Sarge OK, Sarge KD (2004). HSF1 modulation of Hsp70 mRNA polyadenylation via interaction with symplekin. J. Biol. Chem..

[28] Guo Y (2001). Evidence for a mechanism of repression of heat shock factor 1 transcriptional activity by a multichaperone complex. J. Biol. Chem..

[29] Sima S, Schmauder L, Richter K (2019). Genome-wide analysis of yeast expression data based on a priori generated co-regulation cliques. Microb. Cell.

[30] Schmauder, L., Richter, K. hsp-90 and unc-45 depletion induce characteristic transcriptional signatures in coexpression cliques of C elegans. *Sci Rep* **11**, 12852, 10.1038/s41598-021-91690-6 (2021). 10.1038/s41598-021-91690-6 PMC8213770 34145311

[31] Wang P, Zhao J, Corsi AK (2006). Identification of novel target genes of CeTwist and CeE/DA. Dev. Biol..

[32] Jovic K (2017). Temporal dynamics of gene expression in heat-stressed Caenorhabditis elegans. PLoS ONE.

[33] Pachkov M, Erb I, Molina N, van Nimwegen E (2007). SwissRegulon: a database of genome-wide annotations of regulatory sites. Nucleic Acids Res.

[34] Stein L, Sternberg P, Durbin R, Thierry-Mieg J, Spieth J (2001). WormBase: network access to the genome and biology of Caenorhabditis elegans. Nucleic Acids Res..

[35] Wang S, Cheng X, Li Y, Wu M, Zhao Y (2018). Image-based promoter prediction: a promoter prediction method based on evolutionarily generated patterns. Sci. Rep..

[36] Demeler B, Brookes E, Nagel-Steger L (2009). Analysis of heterogeneity in molecular weight and shape by analytical ultracentrifugation using parallel distributed computing. Methods Enzymol..

[37] Haslbeck V (2015). The activity of protein phosphatase 5 towards native clients is modulated by the middle- and C-terminal domains of Hsp90. Sci. Rep..

[38] Garrigues, J. M., Tsu, B. V., Daugherty, M. D. & Pasquinelli, A. E. Diversification of the Caenorhabditis heat shock response by Helitron transposable elements. *eLife* **8**, e51139, doi:10.7554/eLife.51139 (2019). 10.7554/eLife.51139 PMC6927752 31825311

[39] Hietakangas V (2003). Phosphorylation of serine 303 is a prerequisite for the stress-inducible SUMO modification of heat shock factor 1. Mol. Cell Biol..

[40] Holmberg CI, Tran SE, Eriksson JE, Sistonen L (2002). Multisite phosphorylation provides sophisticated regulation of transcription factors. Trends Biochem. Sci..

[41] Zelin E, Freeman BC (2015). Lysine deacetylases regulate the heat shock response including the age-associated impairment of HSF1. J. Mol. Biol..

