# Supplementary material for: Binding of the HSF-1 DNA-binding domain to multimeric C. elegans consensus HSEs is guided by cooperative interactions
Source: Sci Rep. 2022 May 28;12:8984. doi: 10.1038/s41598-022-12736-x (PMC9148306; doi:10.1038/s41598-022-12736-x)
Supplement: Supplementary file 1 — Supplementary Figures. [file 41598_2022_12736_MOESM1_ESM.docx]

**Binding of the HSF-1 DNA-binding domain to multimeric *C. elegans* consensus HSEs is guided by cooperative interactions.**

Lukas Schmauder^1^, Siyuan Sima^1^, Amira Ben Hadj^1^, Ricardo Cesar^1^ and Klaus Richter^1,*^

^1^ Center for Integrated Protein Research at the Department of Chemistry, Technische Universität München, Lichtenbergstr. 4, 85748 Garching

^*^ Corresponding author. E-Mail: klaus.richter@richterlab.de, Tel: +49-89-289-13342

**Supplemental Figure 1.**


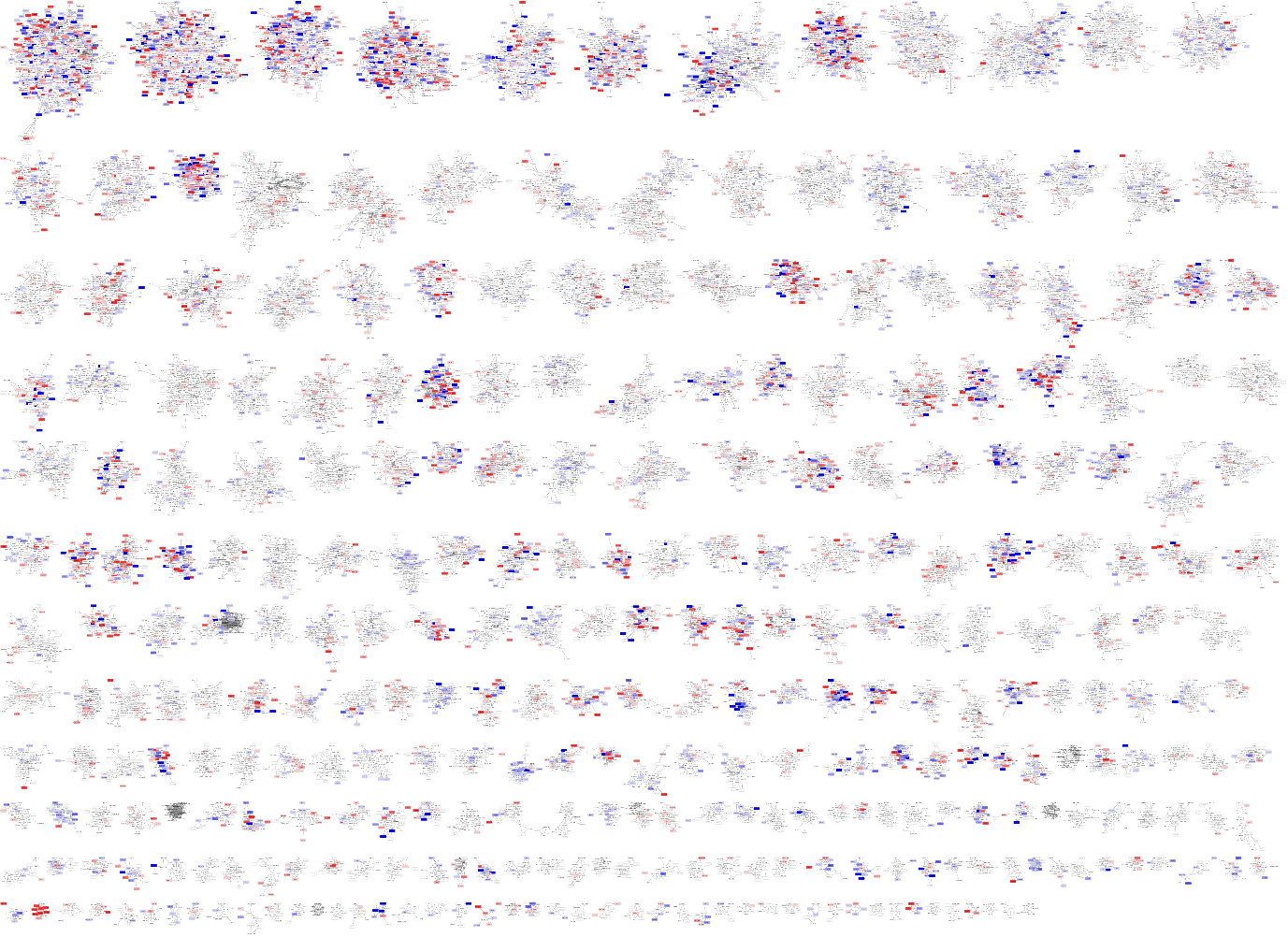


**Genome clustered in 307 expression groups derived from public expression experiments. The color code reflects the heat-shock response as determined by Wang *et al.* (GSE2862).**

**Supplemental Figure 2a**

**
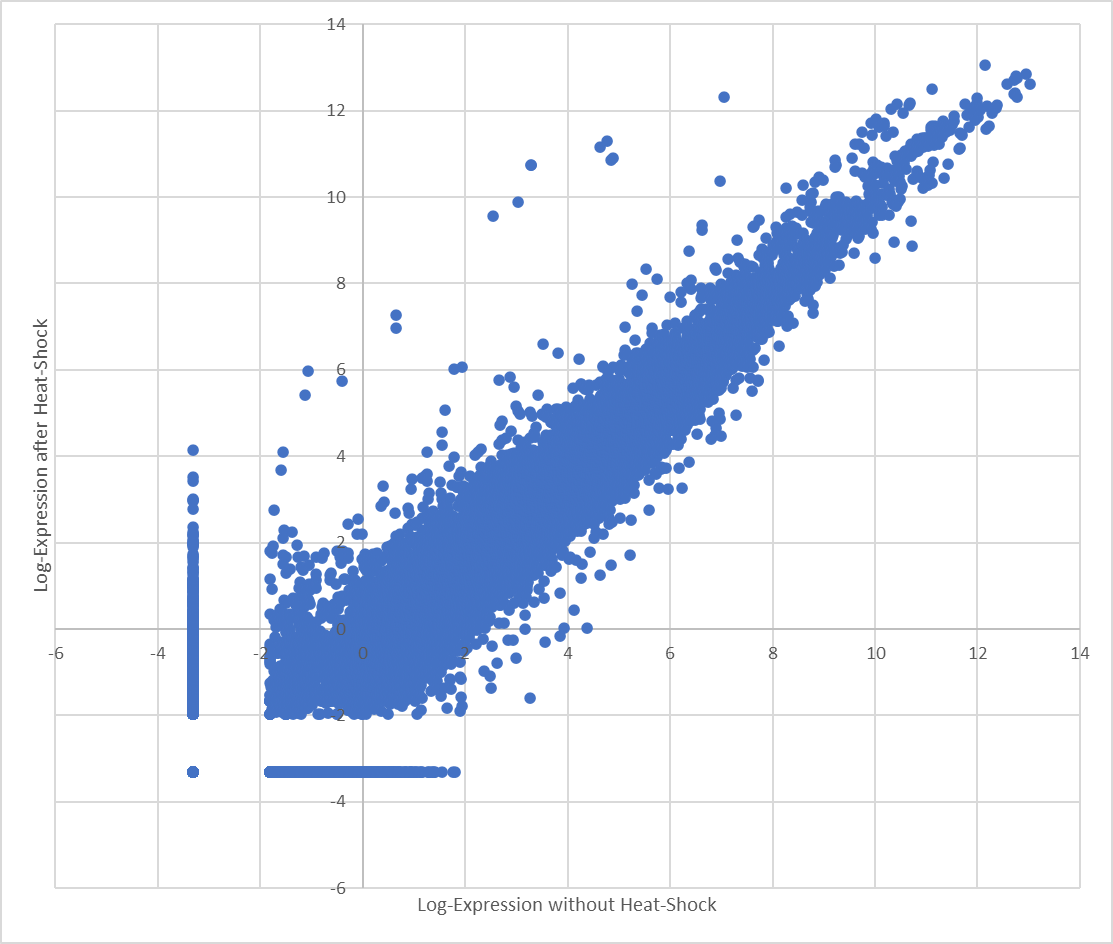
**

**Change in transcriptional expression levels upon heat-shock relative to non heat-shocked specimen, as determined by Brunquell *et al*.**

**Supplemental Figure 2b**

**
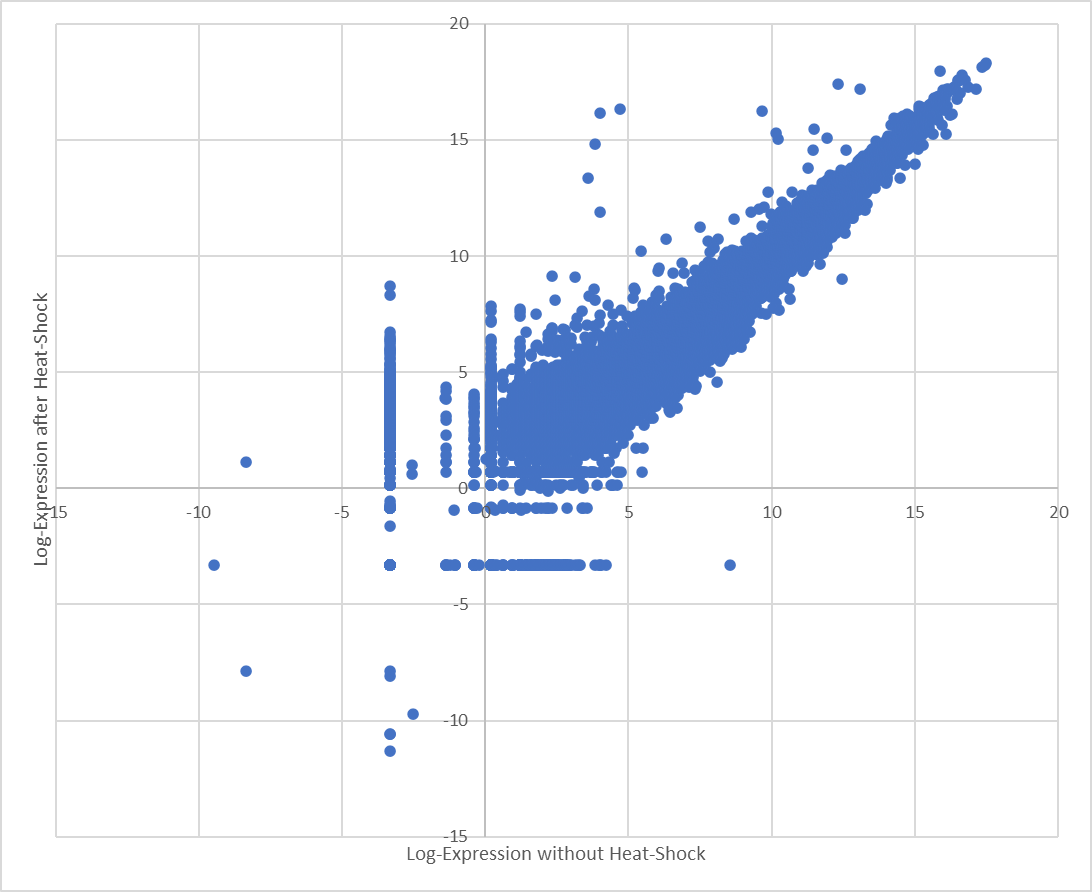
**

**Change in transcriptional expression levels upon heat-shock relative to non heat-shocked specimen, as determined by Li *et al*.**

**Supplemental Figure 2c**

**
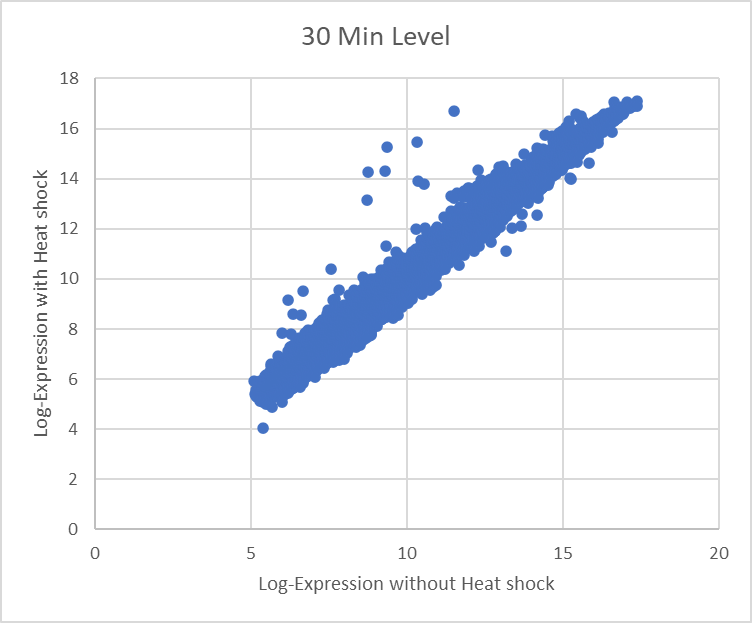
**

**Change in transcriptional expression levels upon heat-shock relative to non heat-shocked specimen, as determined by Jovic *et al.* after 30 minutes of heat-shock.**

**Supplemental Figure 2d**

**
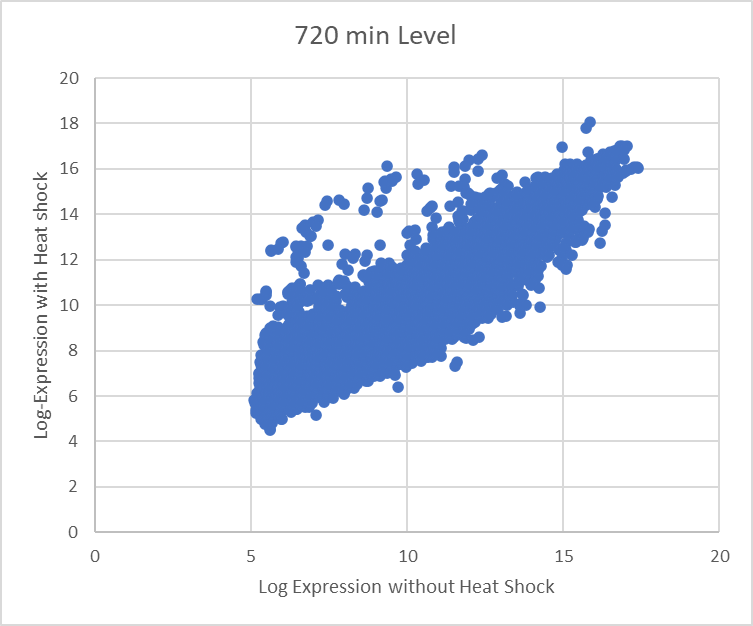
**

**Change in transcriptional expression levels upon heat-shock relative to non heat-shocked specimen, as determined by Jovic *et al.* after 720 minutes of heat-shock.**

**Supplemental Figure 3a**


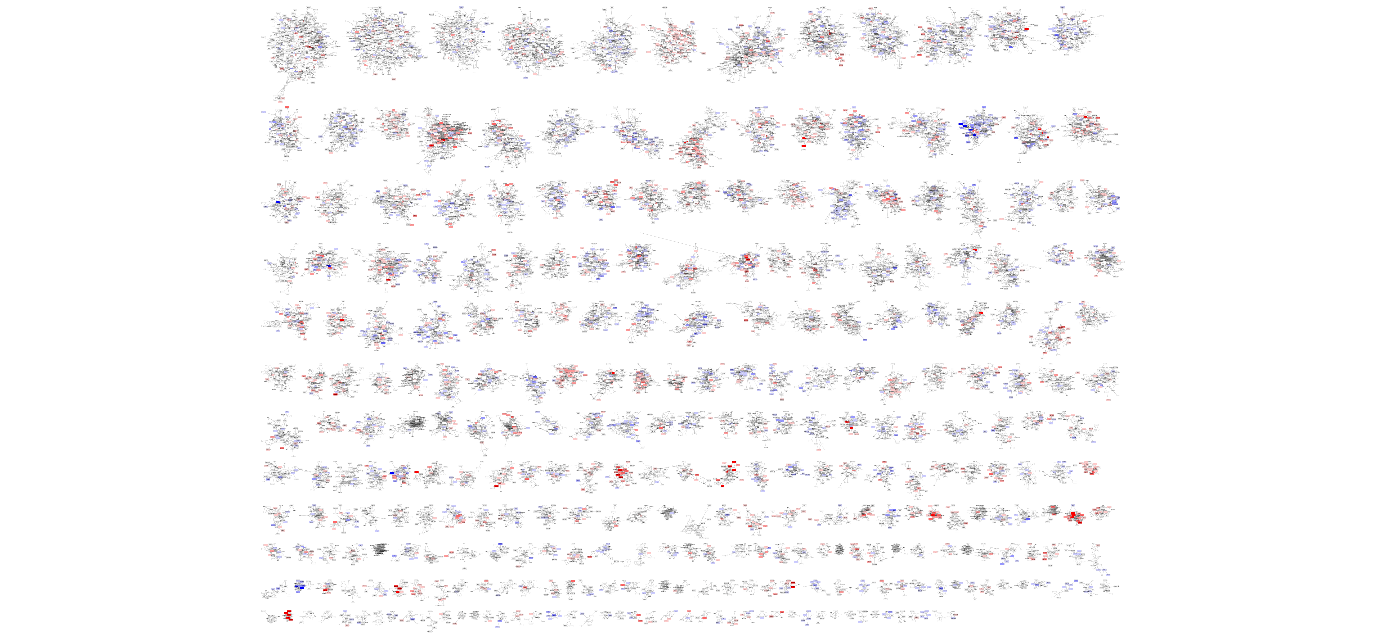


**Genome clustered in 307 expression groups derived from public expression experiments. The color code reflects the heat-shock response as determined by Jovic *et al.* after 30 minutes of heat-shock.**

**Supplemental Figure 3b**


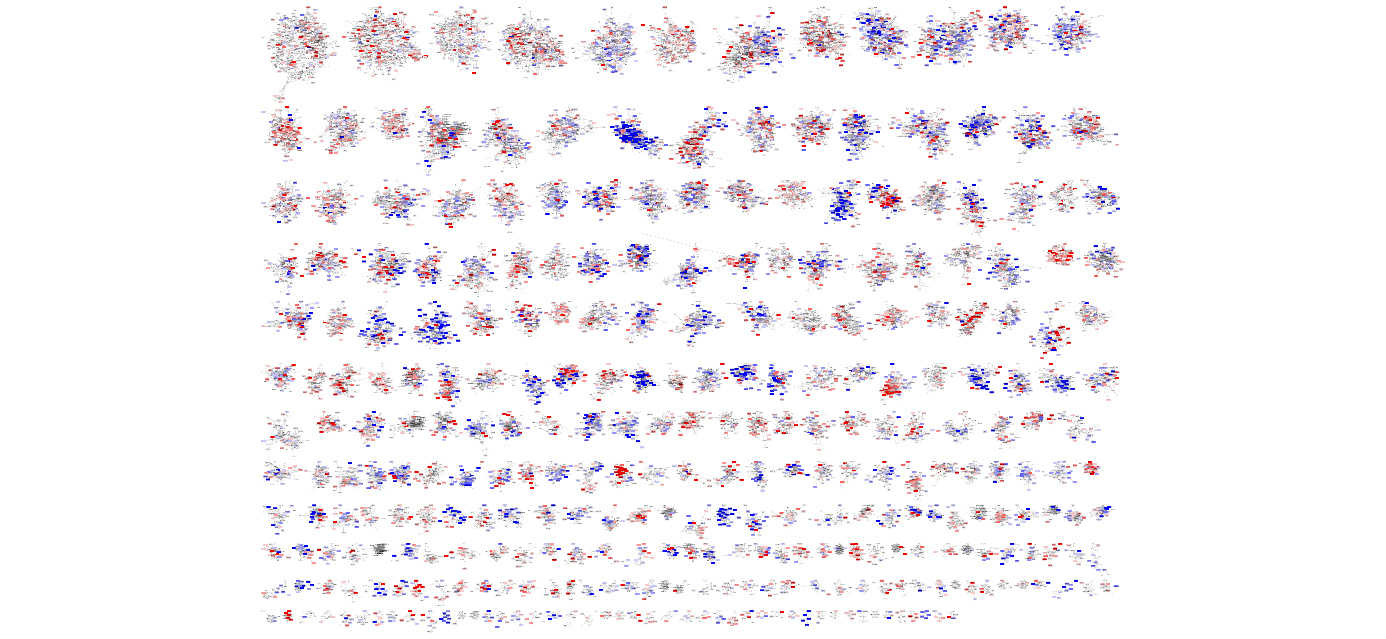


**Genome clustered in 307 expression groups derived from public expression experiments. The color code reflects the heat-shock response as determined by Jovic *et al.* after 720 minutes of heat-shock.**

**Supplemental Figure 4**

**
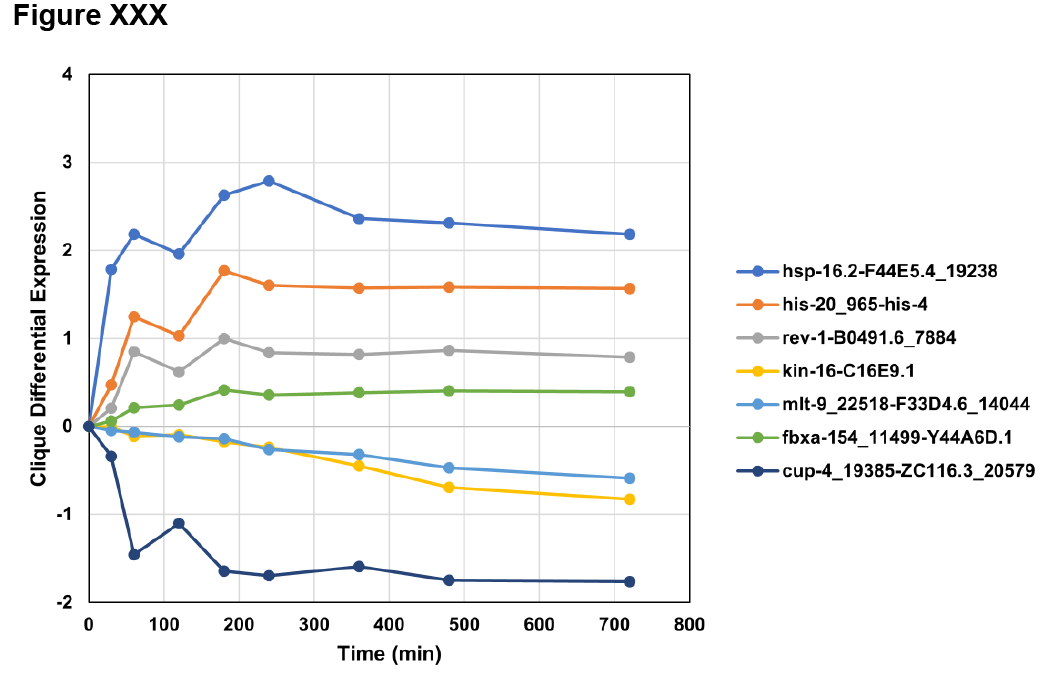
**

**Clique kinetic during heat-shock conditions, based on public expression experiments performed by Jovic *et al*. over a time-course of 720 minutes.**

**Supplemental Figure 5a.**


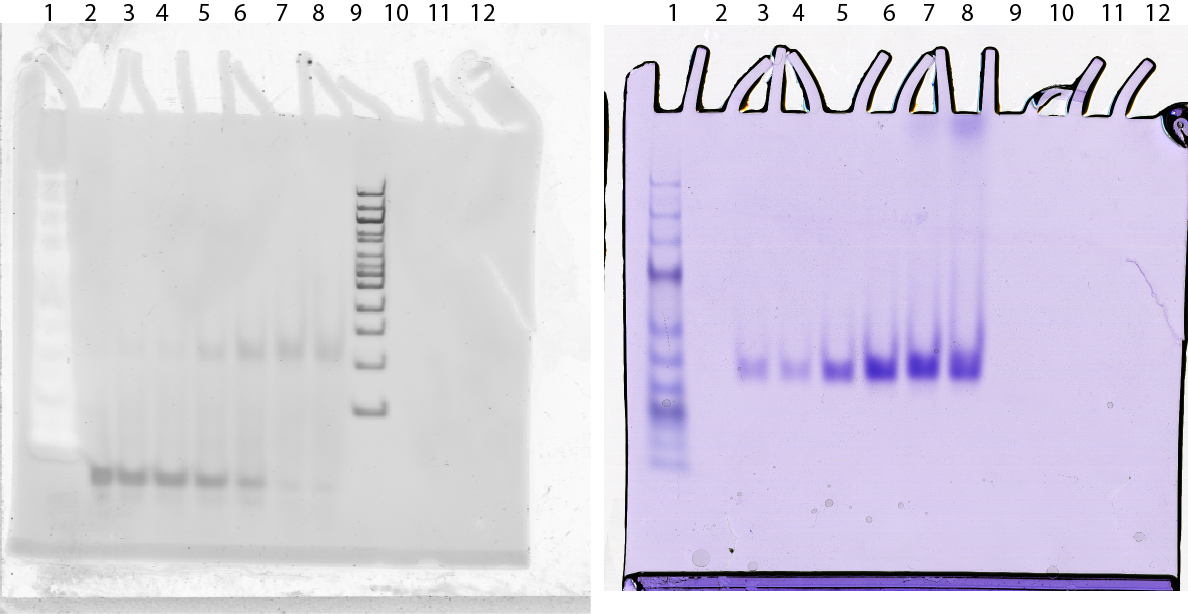


**Titration of HSF-1 DBD to the promotor F44E5.4, ranging from a 1,5-15-fold excess of HSF-1 (2-8)**

**DBD Left gel : DNA stain**

**Right gel : Coomassie blue stain**

1 : Protein marker

2 : F44E5.4

3 : F44E5.4 + 1.5x HSF-1 DBD

4 : F44E5.4 + 3x HSF-1 DBD

5 : F44E5.4 + 5x HSF-1 DBD

6 : F44E5.4 + 7x HSF-1 DBD

7 : F44E5.4 + 10x HSF-1 DBD

8 : F44E5.4 + 15x HSF-1 DBD

9 : DNA marker

**Supplemental Figure 5b.**

**
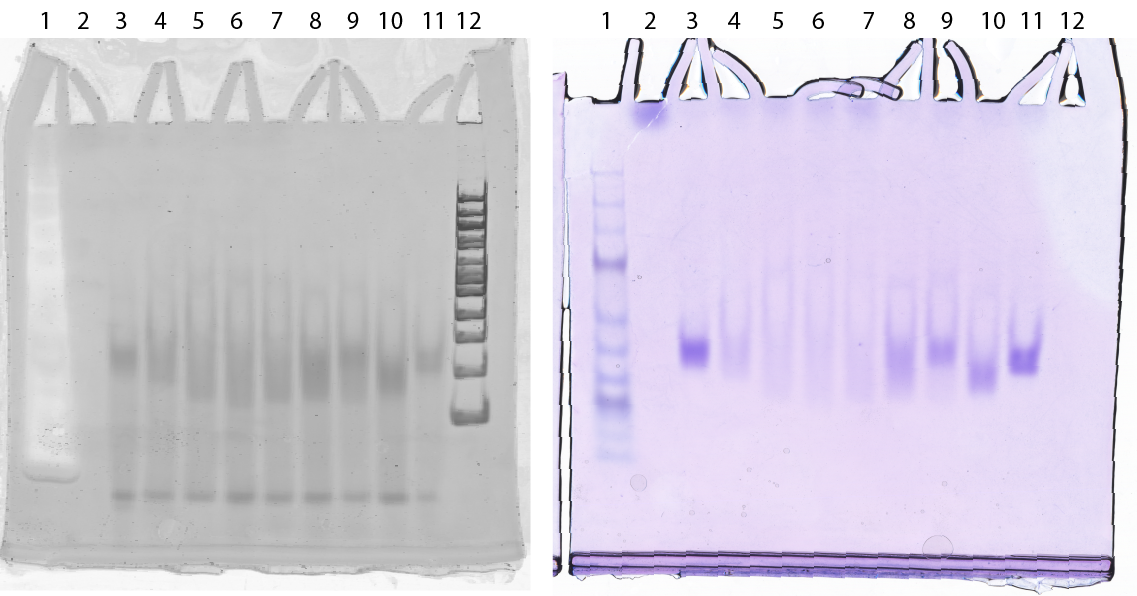
**

**Comparison of selected DNA promotors sequences as indidcated (3-11), each added to the HSF-1 DBD.**

**Left gel : DNA stain**

**Right gel : Coomassie blue stain**

1 : Protein marker

2 : HSF-1 DBD

3 : HSF-1 DBD + Dnj12

4 : HSF-1 DBD + Dnj13

5 : HSF-1 DBD + Bag1

6 : HSF-1 DBD + Unc23

7 : HSF-1 DBD + Hsp1

8 : HSF-1 DBD + Hsp70

9 : HSF-1 DBD + Hsp16.2a

10 : HSF-1 DBD + Hsp16.2b

11 : HSF-1 DBD + F44E5.4

12 : DNA marker
